# Incorporating vertical transmission into mechanistic modeling of West Nile virus for optimized control in Germany

**DOI:** 10.1038/s41598-026-58371-8

**Published:** 2026-07-18

**Authors:** Oliver Chinonso Mbaoma, Stephanie Margarete Thomas, Beierkuhnlein Carl

**Affiliations:** 1 Department of Biogeography, University of Bayreuth, Germany, Bayreuth, Germany; 2 Bayreuth Center for Ecology and Environmental Research, BayCEER, University of Bayreuth, Germany, Bayreuth, Germany

**Keywords:** Vertical transmission, Mosquito-borne disease, West Nile virus, Mosquito control, Epidemic model, Process-based model, Diseases, Ecology, Ecology, Zoology

## Abstract

West Nile virus is an arbovirus transmitted by infected *Culex* mosquitoes to birds, horses, and humans. Since its first recorded case in Germany in 2018, WNV has been endemic in several parts of Germany. Globalization, ecosystem modification and climate change have been identified as significant drivers of the arbovirus spread. However, vertical transmission of pathogens from adult female to their progeny might alter the transmission dynamics. In this study, we designed a process-based epidemic model incorporating climatic variables and epidemiological parameters that replicated mosquito populations and pathogen transmission between mosquito vectors and hosts, explicitly accounting for the role of vertical transmission. In addition, we evaluated the efficiency of biological, chemical, and mechanical strategies for mosquito population control. Our findings revealed that while vertical transmission marginally influenced West Nile virus transmission, the impact was more pronounced in endemic regions, signaling a relationship between vertical transmission and West Nile virus persistence. Among control methods implemented, the biological control method performed optimally and emerged as the preferred method over mechanical and chemical methods, respectively. This model provides an understanding on the impact of vertical transmission in mosquito-borne disease epidemiology and provides insights into the efficiency of mosquito control strategies which would support optimized implementation of mosquito management programs.

**Supplementary Information:**

The online version contains supplementary material available at 10.1038/s41598-026-58371-8.

## Introduction

### West Nile virus infection

West Nile virus (WNV) infection is a viral mosquito-borne zoonosis caused by a single-stranded RNA virus from the Flaviviridae family^1^. Transmission typically occurs between mosquitoes, amplifying hosts (certain bird species), and dead-end hosts (humans and horses) with only mosquitoes and amplifying hosts playing an active role in maintaining the enzootic cycle^2^. About 80% of humans infected after being bitten by infected mosquitoes show no symptoms, while approximately 20% of infected humans develop fever, headache, nausea, vomiting, or occasionally skin rash and swollen nymph nodes^3^. Severe neuroinvasive WNV infection symptoms occur in less than 1% of infected humans which may include headache, high fever, stiff neck tremor, convulsion, muscle weakness and paralysis^4^. While certain birds with high titer show no clinical signs, others like raptors and corvids may even die from infection, and 8% of infected horses develop neurological diseases^5^.

Globally, WNV infection is a mosquito-borne disease of public health concern, with cases reported with outbreaks in every continent^1^. This spread has been attributed to changes in climatic and ecological conditions, which support environmental suitability for mosquito population establishment, pathogen replication, and disease transmission^2^. Also, change in seasonal migration pattern of amplifying host birds have also been identified as a possible cause of introduction of the virus to new geographical locations, as outbreaks have been identified on major migratory routes^6^.

Although different lineages of WNV have been circulating in Europe since 1996, the first autochthonous cases were isolated in 2018 from birds and horses in eastern Germany, with the federal states of Saxony, Saxony-Anhalt, Brandenburg, and Berlin recording the highest number of cases^7^.

### Integrating horizontal and vertical transmission for modeling infection prevalence

**Fig. 1:**
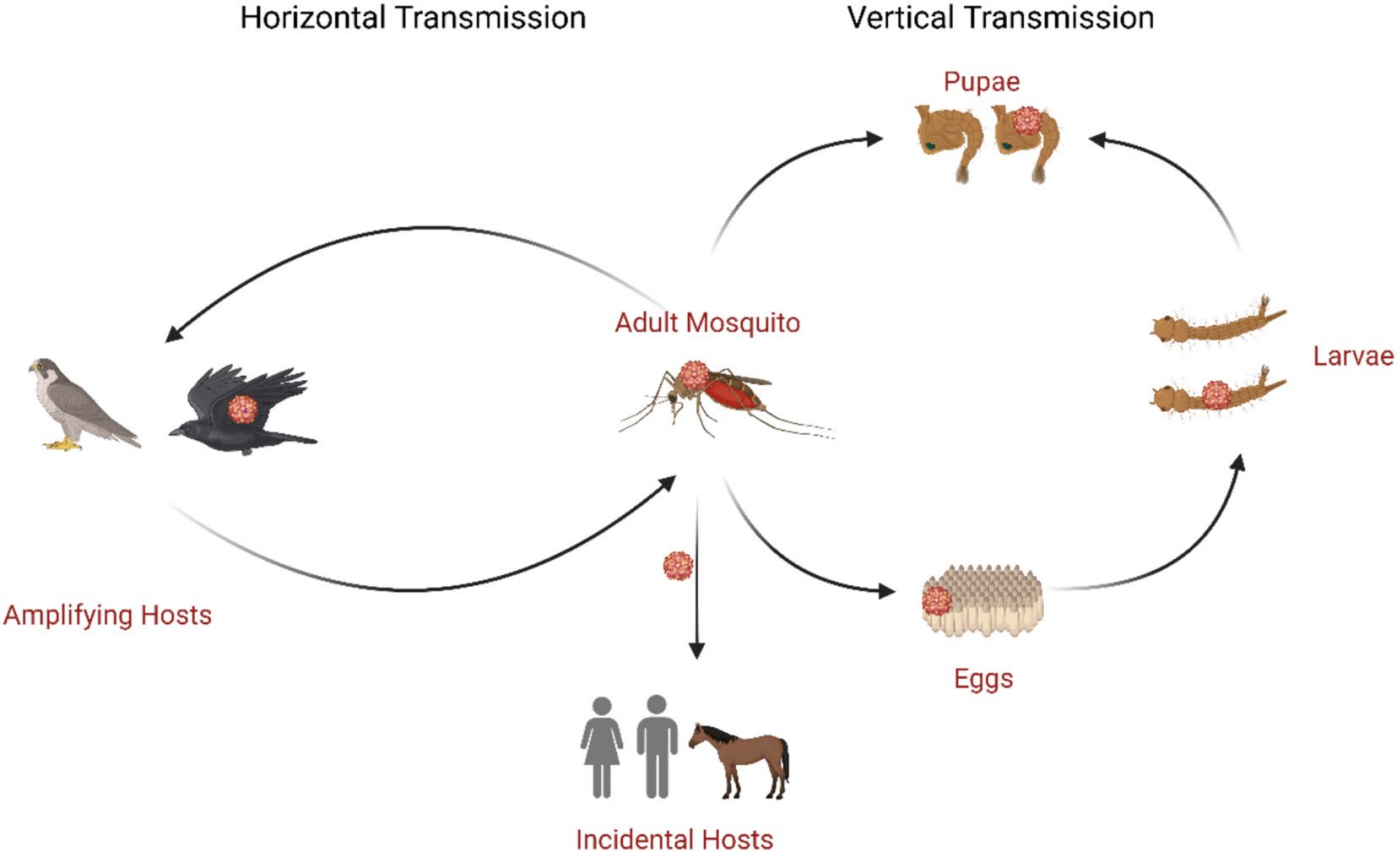
Transmission cycle of West Nile virus showing horizontal and vertical pathways^11^. Transmission of the virus occurs between mosquitoes and competent host birds. Red circles on birds, mosquito eggs, larvae, pupae and adult indicate pathogen infection. Incidental hosts (dead-end hosts) can also become infected but do not support amplification or cross infection. Additionally, adult female mosquitoes can infect their progenies through vertical transmission.

While ecosystem modification, climate change, and urbanization have been identified as key drivers of mosquito-borne diseases^8^, vertical transmission of pathogens from adult mosquitoes to their juvenile may further alter pathogen transmission dynamics^9,10^ (Fig. 1).

Important arboviruses such as Japanese Encephalitis virus, St. Louis Encephalitis virus, Tick-borne encephalitis virus, Chikungunya virus (CHIKV), dengue virus (DENV), Zika virus (ZIKV), and WNV have been found to be transmitted vertically in natural and laboratory conditions^12,13^. Vertical transmission (VT) rates vary among vector species and across virus taxa^14^. For instance, field studies of VT rates for *Aedes albopictus* (*Ae. albopictus*) have reported minimum infection rates of 0.76 for ZIKV and 47.6 for DENV, while minimum infection rate of 3.5 were reported for WNV in *Culex pipiens* (*Cx.pipiens*)^14^. These variations are further influenced by vector population size and dynamics, local temperature fluctuations, the gonotrophic cycle, and mosquito age^15^. VT, which has also been reported by several studies to occur in multiple mosquito species for several arbovirus genera is seen as a possible mechanism of maintaining arbovirus circulation within mosquito population enabling them to survive even during inter-epidemic periods^16–20^. VT of WNV has been identified to occur repeatedly from naturally infected adult *Culex* mosquitoes to their progenies, and suspected to serve as an overwintering reservoir for WNV in South Moravia, Czech Republic, Vienna Austria and California, in the United States^16–18,21–23^.

Given that VT of arboviruses has been confirmed in field and laboratory settings, several modeling studies have integrated the effect of VT in epidemiological models for different mosquito species and arbovirus genera including WNV^14^. Despite evidence of vertical transmission (VT) of WNV in *Cx pipiens* species, only two modeling studies have accounted for it compared with thirteen empirical studies^14,24,25^. Despite being compartmental models which leverage on mechanistic processes, both models were only state structured, giving room for homogeneous mixing, which assumes that every mosquito in the population is infectious, introducing bias into cross-infection process between vector mosquitoes and host^26,27^. Additionally, only a single bird was considered as host, which is contrary to the WNV transmission cycle, which involves multiple bird species typically migratory and residential in nature depending on their migratory status^28,29^. These models were characterized by aggregated results for a large spatial location reported in temporal graphs, lacking spatial and temporal clarity. Also, results from these studies were not fitted or validated with any real data. Regardless of findings from a study by Ferreira-de-Lima et al.^19^ on the existence of a link between endemism and VT of arboviruses as seen in the Americas, this submission has not been explored in other regions where VT has been reported.

To address these challenges, it was necessary to integrate a VT into a WNV epidemic model in addition to horizontal transmission. It was also crucial to understand the impact of VT in WNV infection outbreak dynamics with spatial and temporal clarity, and its role in sustaining WNV infection in geographical locations where WNV is endemic. Despite being reported to occur at negligible rates, we intend to explore the impact of VT on WNV infection epidemiology and its relationship with endemicity in Germany.

### Mosquito control measures

Mosquito control strategies have been implemented in mathematical models^30,31^. The development of an efficient and effective mechanical, chemical or biological control measure requires adequate knowledge about the importance of climatic and ecological factors for specific mosquito species^32,33^. Abiotic site conditions such as temperature, precipitation, or humidity together with biotic interactions such as predation and competition are some of the factors necessary for variation in mosquito population trends. Mechanical, chemical and biological methods have been applied successfully to control mosquito population^32^.

*Cx. pipiens* which is the most important vector of WNV in Europe has been successfully controlled by mechanical methods, involving modification or removal of habitat and breeding sites, chemical control methods, involving insecticides, and biological control, where larvicides and antagonistic bacteria are used^30,34^. In Europe, the use of chemical methods involving application of insecticides are only approved in the event of a disease outbreak whereas bioagents such as larvicides are widely available in the European market and have been often used^35^.

To the best of our knowledge, vector control practices crucial to support efficient implementation of mosquito-borne disease control efforts have not been considered in WNV epidemic models which accounted for the impact of VT^24,25^. This is regardless of the fact that VT has been implicated in hampering the efficiency of mosquito-borne disease outbreak control efforts, as seen in a study by Murillo et al.^36^ reporting difficulties and increased cost of outbreak control with an increase in VT rate.

Leveraging on advances in computational biology, mathematical epidemiology and geospatial data integration, we developed an epidemiological model for estimating transmission risk of WNV integrating VT, and optimal control measures across NUTS3 (Nomenclature of Territorial Units for Statistics) spatial scale in Germany. Model simulated risk maps were validated against WNV infection cases publicly available at the TSIS platform. We used publicly available information on the impact of habitat modification, chemical and larvicide diffusion rates to estimate the impact of habitat removal, use of insecticides, and biological control approaches. We explored the impact of each control measure in terms of WNV infection control across Germany and identified the most efficient method to ensure optimization of control efforts.

## Results

### WNV infection risk with vertical transmission

**Fig. 2:**
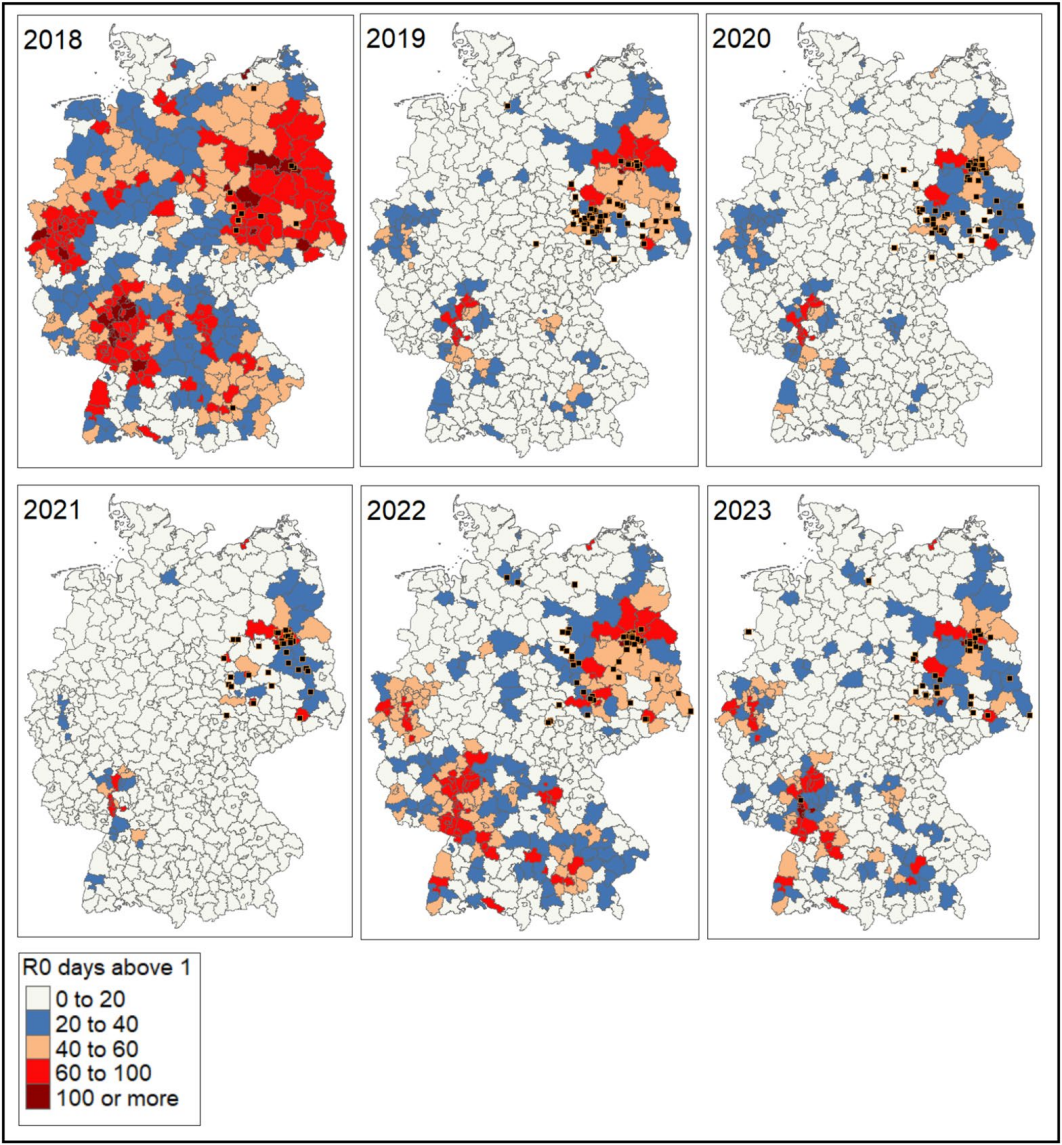
West Nile transmission risk simulated using WNV-VT model. Number of days when WNV Basic Reproductive Number (R_0_) equals or is above 1 representing the onset of transmission risk when accounting for vertical transmission reported at NUTS3 (Nomenclature of Territorial Units for Statistics) spatial scale across Germany between 2018 and 2023. The black dots represent birds case data from FLI^37^.

We simulated mosquito population structure with their infection states and assessed spatial variation in WNV risk across Germany aggregated at NUTS3 spatial scale, using a mechanistic epidemic model for two scenarios; one with VT (WNV-VT model) and the other without VT (WNV model). The simulated WNV infection risk at the NUTS3 administrative level across Germany from 2018 to 2023, correlated with observed WNV cases, with 2018 standing out due to the heatwave across Europe that triggered the onset of WNV transmission in Germany (Fig. 2). Areas in the east, south-west and far west of Germany are recognized as clear hotspot regions, while south-eastern areas are added as risk areas in some years.

**Fig. 3:**
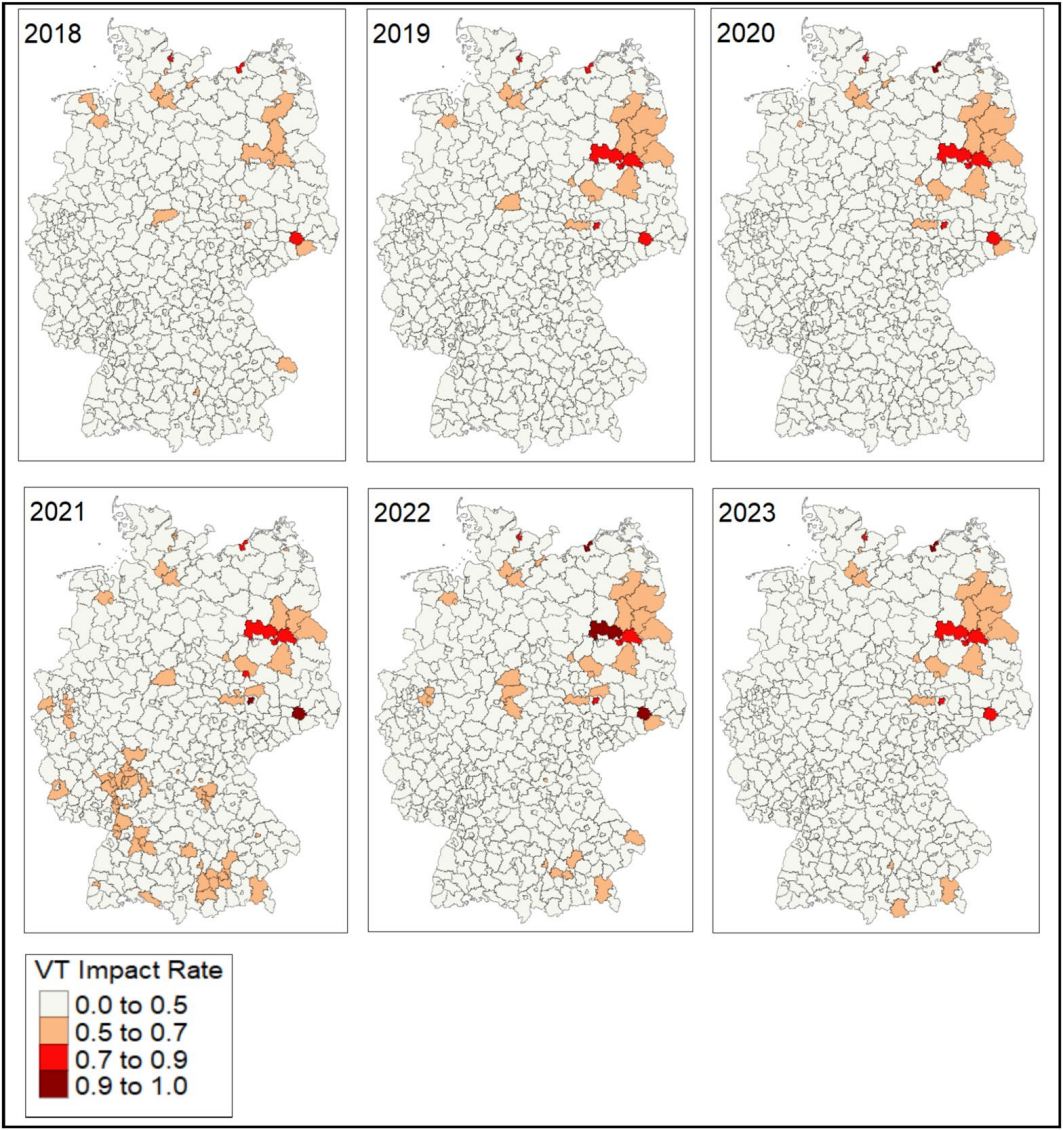
Vertical transmission impact rate on West Nile virus transmission risk across Germany between 2018 and 2023. Vertical transmission impact rate on West Nile virus transmission risk was derived by comparing the difference between the normalized transmission risk simulated using the WNV-VT and the naïve WNV modeling framework.

VT impact rate on WNV transmission risk across Germany was assessed by calculating the normalized difference between WNV transmission risk simulations with the WNV-VT model which incorporates VT (Fig. 2) and the naïve WNV model (see Mboma et al.^27^) from 2018 to 2022 (Fig. 3). Results revealed VT impact rates of above 0.5 as the threshold for identifying locations where WNV has become endemic, with consistent WNV infection occurrences since the virus was first isolated in 2018. Notably, Havelland in Brandenburg, Berlin, Dresden, and Leipzig exhibited VT impact rates of 0.7 and above, correlating with substantial WNV infection incidences in these areas.

**Fig. 4:**
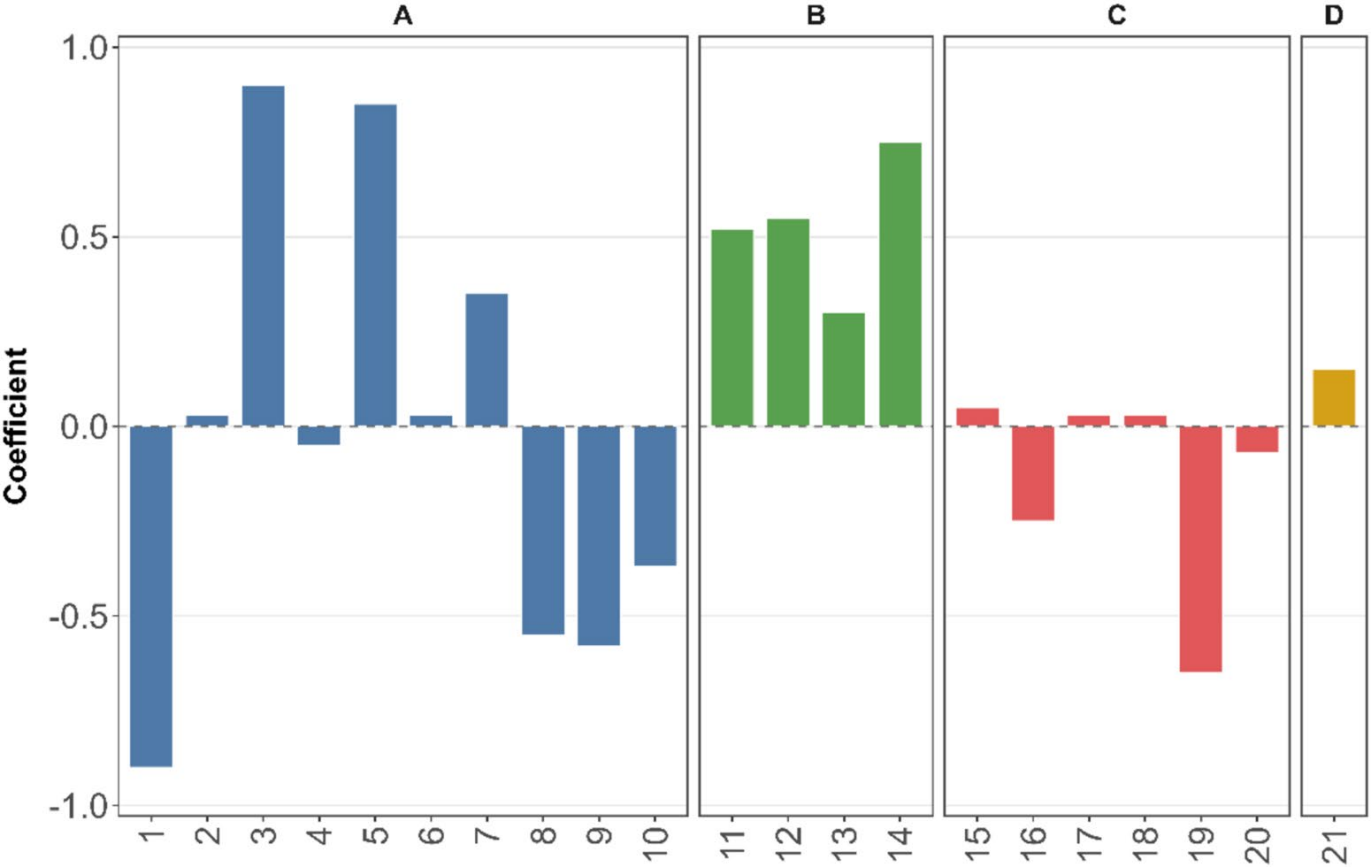
Sensitivity analysis evaluating the impact of selected model parameters on simulated ${R}_{0}$ rates using the PRCC approach. Panels show (**A**) blue bars represent mosquito life-history traits (parameters 1–10: egg mortality, pupae mortality, sex ratio, adult mortality, larvae mortality, oviposition rate, adult development rate, host-seeking rate, risky behavior mortality, mortality at emergence); (**B**) green bars represent transmission probabilities (parameters 11–14: mosquito to residential birds, mosquito to migratory birds, residential birds to mosquito, migratory birds to mosquito); (**C**) red bars represent virulence in birds (parameters 15–20: infectious rate of resident birds, removal rate, death rate due to infection for both resident and migratory birds); and (**D**) yellow bar represents vertical transmission (parameter 21: vertical transmission rate from adult mosquitoes to offspring).

Several parameters were identified to have reasonable effect on infection transmission rate (R_0_) after sensitivity analysis was carried out using the partial rank correlation coefficient approach (Fig. 4). Sensitivity analysis results revealed that R_0_ was more sensitive to VT rate than to variables 2, 4, and 5, which are associated with mosquito life-history traits, as well as variables 15, 17, and 18, which relate to virulence in birds. However, the influence of the VT rate was still less pronounced compared to parameters related to pathogen transmission probability.

### Model result evaluation

**Fig. 5:**
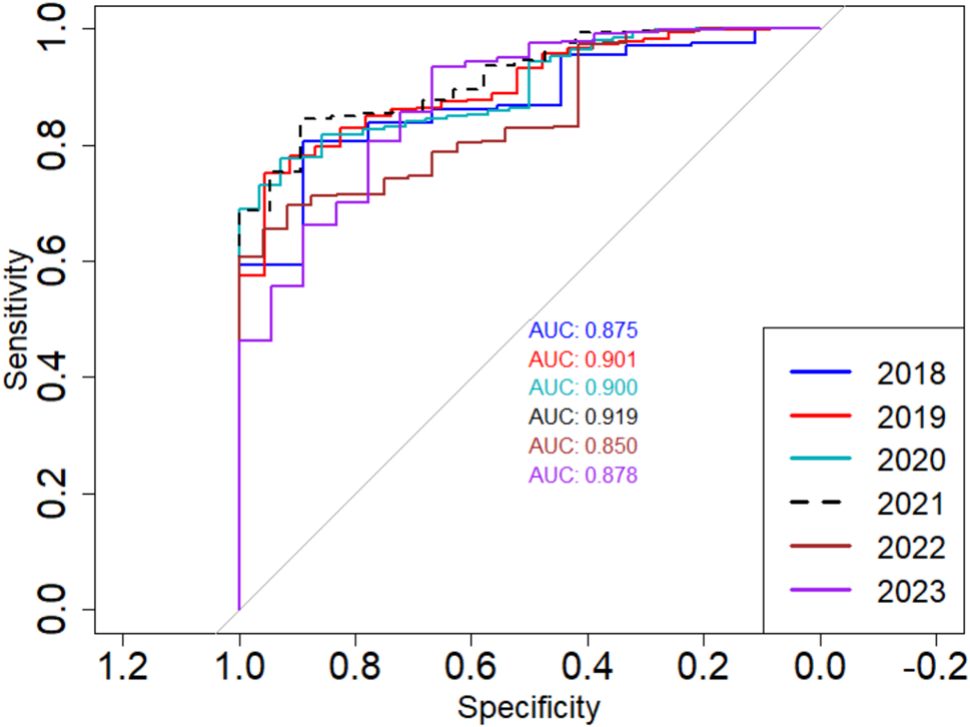
Evaluation of the WNV model with vertical transmission (WNV-VT) using AUC scores computed from ROC curves over six years (2018–2023). Simulated WNV risk values were compared with reported animal infection cases across Germany to assess model performance.

Simulated risk maps from the WNV-VT model were compared with daily WNV infection notification data in birds from the TSIS platform. We evaluated the model’s performance spatially by computing the area under the curve (AUC) values derived from Receiver Operating Characteristics (ROC) curves, which enabled us access ability to accurately simulate spatial risk of WNV infection. The evaluation was premised on comparing model simulated risk maps to observed WNV infection cases in animals reported across Germany over a six-years period from 2018 to 2023 (Fig. 5).

### Mosquito control strategies and control efficiency analysis

**Fig. 6:**
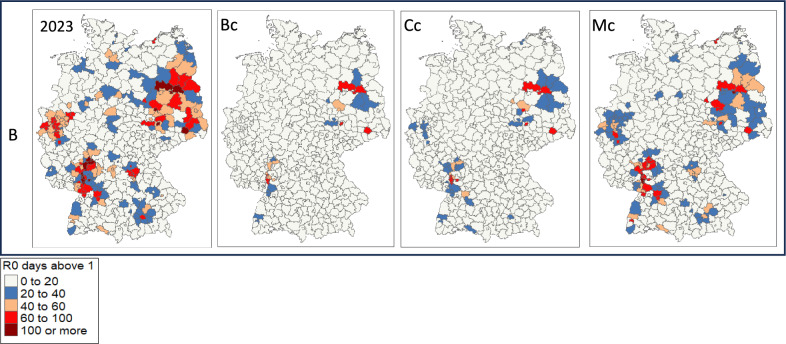
Impact of mosquito control strategies (mechanical, biological and chemical control) on the WNV transmission risk (Number of days when WNV basic reproductive number (R_0_) equals or is above 1) in 2023 (**B**). Maps labelled **Bc** represent WNV infection risk with biological control methods applied, **Cc** represents chemical control while **Mc** represents mechanical control method. (Additional results for 2018–2022 have been documented (Fig. S1). The black dots represent birds case data from FLI^37^.

We evaluated the impact of multiple control strategies and conducted an efficiency analysis to quantify the effectiveness of each intervention. The WNV-VT model assumes that mechanical, biological, and chemical control strategies reduce the vector population, thereby limiting the potential for WNV outbreaks. First, we simulated R_0_ rates without any control method and then applied each control method using the extended WNV epidemic model (Fig. 6). The impact of mosquito control strategies reflected on the outcome of infection transmission risk simulated by our epidemic model across Germany.

**Fig. 7:**
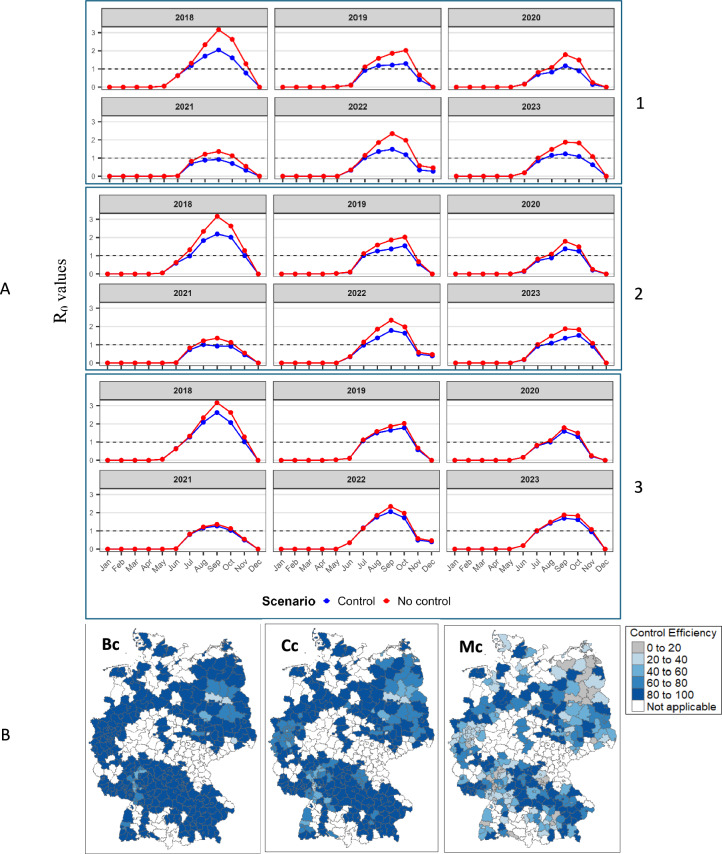
Impact and efficiency of control methods on West Nile virus (WNV) infection risk in Germany. Section **A** shows seasonal impact of control methods from 2018–2023, with the red line representing the period before implementation of control measures, while the blue line indicates the period after implementation. Panels illustrate the effects of different intervention strategies: (1) biological control, (2) mechanical control, and (3) chemical control methods. Section **B** shows spatial control efficiency of each control methods, illustrating the percentage reduction in the infected host population attributted to each control strategy. (Bc) biological control, (Mc) mechanical control, and (Cc) chemical control methods.

The transmission risk (R_0_) was influenced by different mosquito control strategies as shown in (Fig. 7A) between 2018 and 2023, differences between the trend lines for R_0_ rates were implemented before and after control. Comparing the periods before and after the implementation of control measures, a reduction in WNV transmission risk was observed across all intervention strategies. During the peak infection period (August to October), the biological control method demonstrated the highest effectiveness, reducing transmission by up to 34%. The chemical control method also contributed to a noticeable decline, with an approximate reduction of 23% in infection risk. In contrast, the mechanical control method showed a more limited effect, with a reduction of about 10%. Overall, the magnitude of reduction varied among the intervention strategies, with biological control showing the strongest impact during peak seasonal transmission. This effect was also sustained throughout the year, as reflected in the seasonal control impact patterns documented in the analysis. (see Supplementary file, Fig. S1).

The efficiency of control strategies implemented in the WNV-VT model varied spatially across Germany (Fig. 7B). This outcome is necessary to aid in the process of selecting the most efficient, and cost-effective method for mosquito-borne disease control effort in each “Landkreis” (district), state, or region across Germany. Spatially, biological control which involved the use of larvicides to eliminate mosquito larvae was the most effective for all the years. Chemical method which entails eliminating adult mosquitoes with the use of insecticides performed above par with efficiency levels between 40 and 100% but was not as efficient as the biological method with efficiency levels between 60 to 100%. Habitat removal which was related to mechanical control was the least efficient across Germany, with many locations showing only low efficiency values between 0 to 20%

## Discussion

Findings from our study indicate that despite occurring in rates deemed negligible, VT of pathogens can affect infection transmission rates. Results from the research identified a link between VT impact rate and endemicity of WNV infection in several parts of Germany where WNV infection has been endemic. This research also highlights the importance of incorporating VT of epidemic models to account for other mechanisms used by mosquitoes to sustain pathogens in an environment. We were also able to implement biological, chemical, and mechanical control methods. Findings from these implementations identified biological control methods as more effective and efficient across several locations in Germany. These observations provide new insight into the process of designing efficient predictive models needed for mosquito-borne disease monitoring and control.

### Impact of vertical transmission on WNV infection outbreak

Since the first autochthonous cases in 2018, WNV has been endemic in parts of Germany, affecting birds, horses, and humans biennially. While previous studies, including the WNV epidemic model by Mbaoma et al.^27^ have explored outbreak dynamics, the role of vertical transmission (VT) in mosquitoes remains unclear. VT has been documented naturally and experimentally in several mosquito species, including *Cx. pipiens*, the primary WNV vector in Germany and Europe^14,19,38,39^.

Building on these findings, we developed an extended WNV model incorporating VT (WNV-VT) to better capture the impact of VT on WNV transmission dynamics.

Results simulated using the WNV-VT model revealed that VT may have an impact on WNV transmission dynamics in Germany. This is interesting given that the rate of VT considered was only 0.065 or 6.6% estimated between values recorded in studies by Nelms et al.^16^ and Reisen et al.^18^ respectively for *Culex* mosquitoes. This finding also suggests that despite having low rates, VT may impact mosquito-borne disease epidemiology and support their sustenance between inter-epidemic periods, a statement which in agreement with several studies^16–20^.

Sensitivity analysis of the WNV-VT model revealed that although the VT rate had a moderate impact on WNV transmission risk, it was more influential than minimum larval, pupal, and adult mortality, as well as the infection and disease-induced death rates of both resident and migratory birds when equal percentage changes were applied. This finding highlights the importance of VT in mosquito-borne disease epidemiology, despite its conventionally low rate and frequent omission in previous studies^40^.

Spatially, the VT impact rate calculated as the normalized difference between the WNV model and the WNV-VT model varied across locations, consistently highlighting regions where WNV cases have been reported since the onset of transmission in Germany. Impact rates above 0.5 were consistently observed in Eastern Germany for 2018, 2019, 2020, 2022, and 2023, while rates between 0.5 and 1 were recorded in 2021 across Eastern Germany, the Rhine Valley, parts of Northern Germany, and select locations in Bavaria and Nordrhein-Westfalen. WNV infections have been repeatedly detected in most areas where impact rates were greater than 0.5 since the first cases in 2018.These patterns indicates an alignment with previous studies that suggests a relationship between VT, infection transmission rate and endemism^9,19,41^. This is also in agreement with a study by Thongrungkiat et al.^10^ where the onset of a Dengue season was preceded by an elevated VT detection in *Ae. aegypti* larvae collected from the field, signaling the impact of VT on rates of arbovirus outbreak and its significance as an indicator of impending outbreak useful for implementing mosquito control initiatives.

### Control strategies for efficient public health planning

It is important to incorporate control methods into dynamic epidemic models which can support implementation of mosquito control efforts. Several methods have been successfully applied to control mosquito-borne disease outbreaks over the years^30,31^. Besides vaccination and infection treatment, most methods ideally involve strategies that target reduction and elimination of vector population. Mechanical, biological, and chemical control methods have been widely applied in mosquito control programs^25^. Mathematical models are useful tools for optimized simulation of vector-borne disease outbreak, and control with minimal resources^42,43^. These models are typically designed to aid selection of vector control methods for several mosquito species and pathogen genus^30^. They are either generalized for a particular genus with similar functional traits^9,44,45^, while others are specific to certain mosquito species^46–48^. We designed an epidemic model that considered interaction between a single vector and multiple hosts, multiple transmission mechanism and three control methods. This approach was chosen to assess the overall impact of VT, which is often neglected in epidemic models, and assess how effective each control method performed in a transmission regime where VT is included.

*Cx. pipiens* mosquitoes possess the characteristics of environmental plasticity, which enables them to breed in multiple habitats from tree holes, small ponds, semi-permanent water, and ponded vegetations^49^. Although this characteristic makes their population difficult to control, we designed a model that was able to recreate control measures that could be adapted for control of *Cx. pipiens* population and subsequently used to control WNV infection outbreak. These control methods were conceptualized from similar approaches applied by Carvalho et al.^14^.

The biological control method was adapted from replicating the effect of an environmentally friendly approach which involves the use of *Bacillus thuringiensis israelensis* (BTI) based larvicides to eliminate mosquito larvae. The mechanical control method, which involves habitat removal was implemented by reduction of the carrying capacity was the least effective of all methods. This outcome was expected given the environmental plasticity of *Cx. pipiens* mosquitoes and the variety of their habitat choices, making them difficult to control mechanically.

From our results, mechanical control methods performed poorly across locations at risk of WNV infection outbreak for all the years considered. The chemical control method showed a promising but limited effect, reducing the number of exposed mosquitoes marginally. The efficiency of this method could be related to insecticide resistance and chemical diffusion rate which has been accounted for by the continuous function adapted from Carvalho et al.^14^ Due to the sensitivity of chemical-based insecticides to certain environmental conditions, their rate of diffusion in the environment could be affected by several factors, including temperature, humidity, and sunlight, causing them to lose efficacy^50^. Additionally, winged adult mosquitoes have the tendency to evade chemical attack from insecticides by flying to safer locations^51,52^*.* *Culex pipiens* and other adult mosquito species have been reported to develop multiple insecticide resistance mechanisms, including cuticle alterations that reduce insecticide penetration, metabolic resistance through enhanced detoxification of insecticides, and behavioral resistance via avoidance of treated sites^51,53,54^. Studies also suggest that climate change may intensify insecticide resistance by altering mosquito development, metabolism, and activity patterns, thereby reducing reduce the effectiveness of chemical control methods^55^.

Biological method applied outperformed other methods, which brought about a significant reduction in the number of exposed mosquitoes and WNV infection outbreak across Germany for each year. This was expected because biological control targets mosquito larvae before they mature to adults and develop certain abilities that can aid them to survive longer, such as flying and insecticide resistance^56^. Additionally, larvae are confined to aquatic habitats, making them particularly susceptible to larvicide applications^57^. This outcome was also consistent with the results from a previous study by Kroeger et al.^58^ which recorded a significant reduction in the abundance of *Culex pipens* larvae within 3 to 10 days of BTI application in Leipzig. BTI has also been widely applied for mosquito control, tested and designated safe, efficient, cost-effective, and lethal to only target mosquitoes even at high concentration^59^. Notwithstanding, effective application of these control methods for WNV infection, irrespective of their efficiency levels, can be very expensive, given the wide spectrum of *Cx. pipiens* mosquito species habitat choices. Although biological control method using larvicides such as BTI is widely used in Europe and has performed optimally in our study, there are concerns of their side-effect such as affecting non-target organisms, which raises the question of environmental safety and opens the possibility of replacing them with safer methods^35^.

Efficiency analysis is a promising tool for planning mosquito-borne disease control programs. The spatial and temporal distribution of control efficiency can be to optimize and support control method selection. Model simulated control efficiency shows that mechanical control method would be a poor choice to be deployed in areas where WNV infection risk is high and would also perform poorly in locations with VT high impact rate. Control efficiency was also low for all methods and scenarios in locations such as Berlin, Dresden and Leipzig where several WNV cases are isolated yearly and model simulated WNV infection risk is very high. However, an integrated approach could be adopted where several control methods are deployed together to improve efficiency and overcome limitations where they exist. This approach has been successfully deployed to control *Aedes albopictus* population in Southwest of Germany^60^.

### Limitations and conclusion

Despite accounting for the impact of VT in WNV epidemiology across Germany, several limitations remain in the extended model. First, infected juvenile mosquitoes were not explicitly represented across all developmental stages. Including this component would increase model complexity but improve biological realism and capture mosquito life-cycle dynamics more accurately. Second, VT rates were estimated from parameter ranges derived from previous experimental and field studies; however, these parameters may vary geographically due to differences in overwintering rates, land cover, ecosystem modification, and local mosquito ecology. Incorporating region-specific and spatially explicit data would improve model accuracy and transferability. Third, viral strain diversity was not included. As strain variation may influence transmission efficiency and VT rates, future models should incorporate strain-specific parameters to better reflect epidemiological heterogeneity. Finally, only two avian host species were considered, limiting ecological representation of WNV transmission in Germany. Expanding the model to include additional bird species with species-specific competence and abundance would improve ecological realism and predictive performance.

We developed a WNV epidemic model that incorporates the role of VT, a process that has often been overlooked in predictive models of mosquito-borne diseases. VT impact rate computed by comparing WNV model (no VT) and WNV-VT model (With VT) revealed spatial heterogeneity in VT impact across Germany, with higher values observed in regions where WNV cases have been consistently reported, suggesting relationship between VT and endemic circulation of WNV in those locations. Furthermore, evaluation of control strategies indicated that biological control was the most effective intervention, outperforming both chemical and mechanical approaches. The simulated control method efficiency also varied geographically, highlighting differences in intervention performance across regions and providing guidance for selecting context-specific vector control strategies to support public health decision-making.

Overall, the model captures VT-mediated transmission from adult mosquitoes to their progeny and integrates multiple control interventions, revealing distinct spatial patterns in transmission dynamics and control effectiveness. These findings provide further insight into WNV epidemiology, particularly the role of VT in sustaining transmission, and support the optimization of mosquito control programs across Germany.

## Materials and methods

### West Nile virus epidemic model with vertical transmission and control

We previously developed a spatiotemporally explicit epidemic model for WNV infection outbreak at NUTS 3 spatial scale across Germany^27^. The precursor model was based on the integration of *Cx. pipiens* mosquito population model conceptualized from studies by Tran et al.^61^ Ezanno et al.^62^, and an epidemic model for WNV adapted from Laperriere et al.^28^ to develop an inverse-calibrated epidemic model to understand the spatial and temporal dynamics of WNV infection in Germany^27^.

For the new WNV epidemic model with VT and mosquito control, environmental, epidemiological, and bird data with similar spatial dimension were used. Temperature, precipitation, and humidity data were sourced from E-OBS gridded dataset freely available^63^. Human and animal cases of WNV infection, representing epidemiological data, were collected from Robert Koch-institute and TSIS platform of the Friedrich Loeffler Institute^37^. Occurrence data of Hooded Crow and Northern Goshawk identified as important migratory and resident birds that are highly susceptible to WNV and important in sustaining the transmission cycle of the virus were collected from E-bird archive^64^. The temporal dimension of our model simulation was between 2018 and 2023 across Germany.

**Fig. 8:**
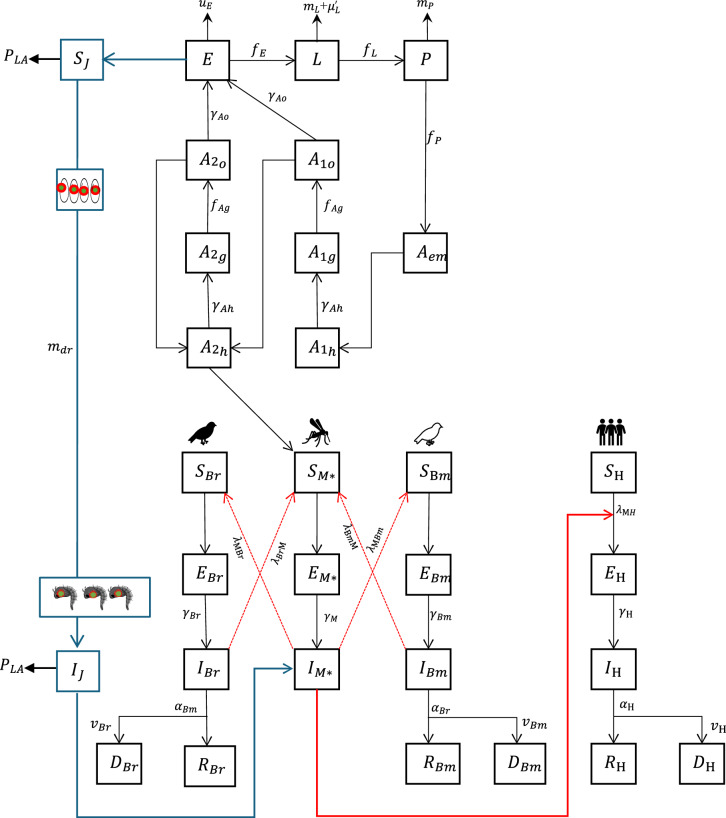
Extended WNV epidemic model: (1) Mosquito population and (2) WNV infection explaining pathogen transmission between mosquito, resident birds, migratory birds and humans. State variables and parameters have been defined in Tables 1, Table 2. All adult mosquito compartments ${(A}_{\mathrm{x}\mathrm{y}}$) and those with asterisk were modulated by adult mortality rate ( ${m}_{A}+{\mu}_{A}^{^{\prime}}$).

Vector population growth was described in ten compartments representing the juvenile and adult stages of mosquito life cycle. Pathogen transmission was explained by eighteen compartments describing the state of health of susceptible mosquitoes, residential birds, migratory birds, and humans. The model was age and stage structured. In addition, we also considered susceptible and infected juveniles supplementing infected mosquitoes, and three control mechanisms via specific parameters to account for each control strategy (Fig. 8).

**Table 1:** State variables and their initial values for WNV Epidemic model integrating VT and mosquito control.

Parameter	Description	Initial values	
$\dot{E}$	Egg	(10^7^ × *A*) /100000	
$L$	Lavae	0	
$P$	Pupa	0	
${A}_{em}$	Emerging adult mosquitoes	0	
${\dot{A}}_{1h}$	New host-seeking mosquitoes	0	
${A}_{1g}$	New gestating mosquitoes	0	
${A}_{1o}$	New ovipositioning mosquitoes	0	
${A}_{2h}$	Old gestating mosquitoes	0	
${A}_{2g}$	Old ovipositioning mosquitoes	0	
${S}_{\mathrm{J}}$	Susceptible juvenile mosquitoes	0	
${I}_{\mathrm{J}}$	Infected juvenile mosquitoes	0	
${S}_{\mathrm{M}}$	Susceptible adult mosquitoes	0	
${E}_{\mathrm{M}}$	Exposed adult mosquitoes	0	
${I}_{\mathrm{M}}$	Infected adult mosquitoes	0	
${S}_{\mathrm{B}\mathrm{r}}$	Susceptible residential birds	${K}_{\mathrm{B}\mathrm{r}}$ × 0.10	
${E}_{\mathrm{B}\mathrm{r}}$	Exposed residential birds	0	
${I}_{\mathrm{B}\mathrm{r}}$	Infected residential birds	0	
${R}_{\mathrm{B}\mathrm{r}}$	Removed residential birds	0	
${D}_{\mathrm{B}\mathrm{r}}$	Dead residential birds	0	
${S}_{\mathrm{B}\mathrm{m}}$	Susceptible migratory birds	${K}_{\mathrm{B}\mathrm{m}}$ × 0.10	
${E}_{\mathrm{B}\mathrm{m}}$	Exposed migratory birds	0	
${I}_{\mathrm{B}\mathrm{m}}$	Infected migratory birds	0	
${R}_{\mathrm{B}\mathrm{m}}$	Removed migratory birds	0	
${D}_{\mathrm{B}\mathrm{m}}$	Dead migratory birds	0	

*A* is the area of study location.

**Table 2:** Parameters for the WNV Epidemic model considering VT and mosquito control. Methods and processes used for calibration has been explained in Mbaoma et al.^27^.

Parameter	Description	Value
$\sigma$	Sex-ratio at emergence	0.5
${\gamma}_{Aem}$	Development rate of emerging adults	0.54
${\gamma}_{Ah}$	Transition rate from host seeking to engorged adult(day − 1)	0.549
${\gamma}_{Ao}$	Minimum transition rate from host seeking to engorged adult(day − 1)	0.33
${\mu}_{e}$	Minimum egg mortality rate(day − 1)	0.015
${\mu}_{l}$	Minimum larvae mortality rate(day − 1)	0.03
${\mu}_{p}$	Minimum pupae mortality rate(day − 1)	0.655
${\mu}_{A}$	Minimum adult mortality rate (day-^1^)	0.04
${\mu}_{em}$	Mortality rate during emergence(day − 1)	0.109
${\mu}_{r}$	Mortality rate related to seeking behavior (day − 1)	0.072
${\mu}_{pr}$	Mortality rate related to predators (day − 1)	0.4
$TD{D}_{Ag}$	Number of degree-days needed for egg maturation	64.4
${T}_{Ag}$	Minimal temperature needed for egg maturation	9.8
${k}_{Lf}$	Standard carrying capacity for larvae (per Km^2^)	31,875
${k}_{Pf}$	Standard carrying capacity for pupae	${k}_{Lf}$*0.97
${k}_{Mf}$	Standard capacity for mosquito	${k}_{Pf}$*0.9
${J}_{v}$	Juvenile infectious rate	0.07
${\gamma}_{\mathrm{B}\mathrm{r}}$	Infectious rate residential birds	0.196
${\gamma}_{\mathrm{B}\mathrm{m}}$	Infectious rate migratory birds	0.285
${\alpha}_{\mathrm{B}\mathrm{r}}$	Removal rate residential birds	0.867
${\alpha}_{\mathrm{B}\mathrm{m}}$	Removal rate migratory birds	0.40
${m}_{\mathrm{B}\mathrm{r}}$	Natural death rate residential birds	0.0005
${m}_{\mathrm{B}\mathrm{m}}$	Natural death rate migratory birds	0.00023
${v}_{\mathrm{B}\mathrm{r}}$	Death rate due to infection for residential birds	0.655
${v}_{\mathrm{B}\mathrm{m}}$	Death rate due to infection for migratory birds	0.103
${b}_{\mathrm{H}}$	Birth rate humans	0.000055
${\gamma}_{\mathrm{H}}$	Infectious rate humans	0.25
${\alpha}_{\mathrm{H}}$	Removal rate humans	0.5
${m}_{\mathrm{H}}$	Natural death rate humans	0.000034
${v}_{\mathrm{H}}$	Death rate due to infection for humans	0.004
${p}_{MBr}$	Probability of transmission from mosquito to residential birds	0.97
${p}_{MBm}$	Probability of transmission from mosquito to migratory birds	0.9
${p}_{MH}$	Probability of transmission from mosquito to humans	0.5
${p}_{BrM}$	Probability of transmission from residential birds to mosquitoes	0.4
${p}_{BmM}$	Probability of transmission from migratory birds to mosquitoes	0.7
${F}_{b}$	Proportion of mosquito feeding on birds	0.25
${F}_{h}$	Proportion of mosquito feeding on humans	0.38

Additional compartments adapted from a study by Chitnis et al.^9^ representing susceptible and infected juvenile mosquito stages respectively, were integrated to represent progenies that were susceptible and eventually infected vertically from their parents. Both juvenile compartments were replenished at a per capita development rate (${m}_{dr}$) and a per capita mortality rate (${P}_{LA}$ ) adapted from Shocket et al.^65^. Infected mosquitoes lay infected eggs at a proportion (${J}_{v}$) estimated from studies by Nelms et al.^16^ and Reisen et al.^18^ respectively where experimental and natural VT of WNV were confirmed in *Cx. pipiens* population.

Mechanical mosquito control strategy was based on habitat modification by reducing the environmental carrying capacity (${k}_{L}$, ${k}_{P}$, ${k}_{M}$) through a coefficient (${H}_{O}$)^30^. Chemical control strategy was implemented via insecticide which targets adult mosquitoes at a rate defined by ${\mu}_{A}^{^{\prime}}$. Biological control strategy implies the use of bioagent such as larvicide to eliminate mosquito larvae at a rate ${\mu}_{L}^{^{\prime}}$.

To implement mechanical control, carrying capacities for our model (${k}_{Lv}$, ${k}_{Pv}$, ${k}_{Mv}$) were denoted in Eq. (1–3).

1 $${{k}_{Lv}{\prime}=k}_{Lv}({H}_{O}/{k}_{Lf})$$

2 $${{k}_{Pv}{\prime}=k}_{Pv}({H}_{O}/{k}_{Pf})$$

3 $${{k}_{Mv}{\prime}=k}_{Mv}({H}_{O}/{k}_{Mf})$$

With ${H}_{O}$ having a minimum value of 0 and a maximum value of 1.

To implement chemical control, efficacy is represented by a value ${\mu}_{A}^{^{\prime}0}$, modulated by a chemical decay function ${e}^{-kt}$. The final equation representing chemical control strategy is given as shown in Eq. (4).

4 $${\mu}_{A}^{^{\prime}}={\mu}_{A,}^{^{\prime}0}{ e}^{-kt}$$

When implemented, the mortality rate of adults was modified as shown in Eq. (5).

5 $${m}_{A}{\prime}={m}_{A}+{\mu}_{A}^{^{\prime}}$$

With ${\mu}_{A}^{^{\prime}}$ having a minimum value of 0 and a maximum value of 1.

For biological control, mortality rate of larvae was defined by a value ${\mu}_{L}^{^{\prime}0}$ which represents the efficacy of the larvicide applied and decayed at a rate ${\prime}{e}^{-kt}$.

The final equation representing biological control strategy was given as shown in Eq. (6).

6 $${\mu}_{L}{\prime}={\mu}_{L,}^{^{\prime}0} {{\prime}e}^{-kt}$$

When implemented, the mortality rate of larvae was modified as shown in Eq. (7).

7 $${{m}_{L}{\prime}+m}_{L}{+\mu }_{L}^{^{\prime}}$$

With ${\mu}_{L}^{^{\prime}}$ having a minimum value of 0 and a maximum value of 1.

For VT, two additional compartments were added to the susceptible mosquito part of the pathogen transmission section, representing susceptible juvenile and infected juvenile mosquitoes (S_J_, I_J_). Susceptible juvenile mosquitoes transited into the susceptible mosquito compartment. Furthermore, infected juvenile mosquitoes transited into the infected mosquito compartment to account for infection through VT.

The overall processes driving mosquito population and pathogen transmission were explained in the coupled Eq. (8), while the process driving pathogen transmission within the population were explained by coupled ordinary differential equations (ODEs) in Eq. (9–12).

8 $$\left\{\begin{array}{c}\dot{E}={\delta}_{M}{\gamma}_{Ao}\left({\beta}_{1}{A}_{1o}+{\beta}_{2}{A}_{2o}\right)-\left({\mu}_{E}+{\delta}_{M}{f}_{E}\right)E\\ \dot{L}={\delta}_{M}{f}_{E}E-\left(({m}_{L}+{\mu}_{L}^{^{\prime}})\left(1+\frac{L}{{k}_{Lv}+{H}_{O})}\right)+{f}_{L}\right)L\\ \dot{P}={f}_{L}L-\left({m}_{P}+{f}_{P}\right)P\\ \dot{{A}_{em}}={f}_{P}P\sigma e\left(-{\mu}_{em}\left(1+\frac{P}{{k}_{Pv}+{H}_{O})}\right)\right)-\left({m}_{A}+{\gamma}_{Aem}\right){A}_{em}\\ {\dot{A}}_{1h}={\gamma}_{Aem}\dot{{A}_{1h}}-(\left({m}_{A}+{\mu}_{A}^{^{\prime}})+{\mu}_{r}+{\gamma}_{Ah}\right){A}_{1h}\\ \dot{{A}_{1g}}={\gamma}_{Ah}{A}_{1h}-\left(({m}_{A}+{\mu}_{A}^{^{\prime}})+{\mu}_{pr}+{f}_{Ag}\right){A}_{1g}\\ \dot{{A}_{1o}}={f}_{Ag}{A}_{1g}-(\left({m}_{A}+{\mu}_{A}^{^{\prime}})+{\mu}_{r}+{f}_{Ao}\right){A}_{1o}\\ \dot{{A}_{2h}}={f}_{Ao}\left({A}_{1o}+{A}_{2o}\right)-(\left({m}_{A}+{\mu}_{A}^{^{\prime}})+{\mu}_{r}+{\gamma}_{Ah}\right){A}_{2h}\\ \dot{{A}_{2g}}={\gamma}_{Ah}{A}_{2h}-(\left({m}_{A}+{\mu}_{A}^{^{\prime}})+{\mu}_{pr}+{f}_{Ag}\right){A}_{2g}\\ \dot{{A}_{2o}}={f}_{Ag}{A}_{2g}-(\left({m}_{A}+{\mu}_{A}^{^{\prime}})+{\mu}_{r}+{f}_{Ao}\right){A}_{2o}\end{array}\right.$$

9 $$\begin{array}{c}\frac{\mathrm{d}{S}_{\mathrm{J}}}{\mathrm{d}t}={P}_{LA}\left({J}_{v}{I}_{\mathrm{M}}\right){A}_{2o}-{m}_{dr}\left(T\right){S}_{\mathrm{J}}\\ \frac{\mathrm{d}{I}_{J}}{\mathrm{d}t}={P}_{LA}\left(\frac{{\varphi}_{v}{I}_{M}}{{A}_{2o}}\right)-{m}_{dr}\left(T\right){I}_{J}\\ \end{array}$$

10 $$\begin{array}{c}\frac{\mathrm{d}{S}_{\mathrm{M}}}{\mathrm{d}t}=\left(-{\lambda}_{BrM}+{\lambda}_{BmM}\right){S}_{\mathrm{M}}{+(A}_{2h}+{S}_{\mathrm{J}})-({m}_{A}+{\mu}_{A}^{^{\prime}}){S}_{\mathrm{M}}\\ \frac{\mathrm{d}{E}_{\mathrm{M}}}{\mathrm{d}t}={(\lambda }_{BrM}+{\lambda}_{BmM}){S}_{\mathrm{M}}-{\gamma}_{\mathrm{M}}{E}_{\mathrm{M}}-({m}_{A}+{\mu}_{A}^{^{\prime}}){E}_{\mathrm{M}}\\ \frac{\mathrm{d}{I}_{\mathrm{M}}}{\mathrm{d}t}={\gamma}_{\mathrm{M}}{E}_{\mathrm{M}}-({m}_{A}+{\mu}_{A}^{^{\prime}}){I}_{\mathrm{M}}\end{array}$$

11 $$\begin{array}{c}\frac{\mathrm{d}{S}_{\mathrm{B}\mathrm{r}}}{\mathrm{d}t}=\left({b}_{\mathrm{B}\mathrm{r}}-\left({b}_{\mathrm{B}\mathrm{r}}-{m}_{\mathrm{B}\mathrm{r}}\right)\frac{{N}_{\mathrm{B}\mathrm{r}}}{{K}_{\mathrm{B}\mathrm{r}}}\right){N}_{\mathrm{B}\mathrm{r}}-{\lambda}_{MBr}{S}_{\mathrm{B}\mathrm{r}}-{m}_{\mathrm{B}\mathrm{r}}{S}_{\mathrm{B}\mathrm{r}}\\ \frac{\mathrm{d}{E}_{\mathrm{B}\mathrm{r}}}{\mathrm{d}t}={\lambda}_{MBr}{S}_{\mathrm{B}\mathrm{r}}-{\gamma}_{\mathrm{B}\mathrm{r}}{E}_{\mathrm{B}\mathrm{r}}-{m}_{\mathrm{B}\mathrm{r}}{E}_{\mathrm{B}\mathrm{r}}\\ \frac{\mathrm{d}{I}_{\mathrm{B}\mathrm{r}}}{\mathrm{d}t}={\gamma}_{\mathrm{B}\mathrm{r}}{E}_{\mathrm{B}\mathrm{r}}-{\alpha}_{\mathrm{B}\mathrm{r}}{I}_{\mathrm{B}\mathrm{r}}-{m}_{\mathrm{B}\mathrm{r}}{I}_{\mathrm{B}\mathrm{r}}\\ \frac{\mathrm{d}{R}_{\mathrm{B}\mathrm{r}}}{\mathrm{d}t}=\left(1-{v}_{\mathrm{B}\mathrm{r}}\right){\alpha}_{\mathrm{B}\mathrm{r}}{I}_{\mathrm{B}\mathrm{r}}-{m}_{\mathrm{B}\mathrm{r}}{R}_{\mathrm{B}\mathrm{r}}\\ \frac{\mathrm{d}{D}_{\mathrm{B}\mathrm{r}}}{\mathrm{d}t}={v}_{\mathrm{B}\mathrm{r}}{\alpha}_{\mathrm{B}\mathrm{r}}{I}_{\mathrm{B}\mathrm{r}}\end{array}$$

12 $$\begin{array}{c}\frac{\mathrm{d}{S}_{\mathrm{B}\mathrm{m}}}{\mathrm{d}t}=\left({b}_{\mathrm{B}\mathrm{m}}-\left({b}_{\mathrm{B}\mathrm{m}}-{m}_{\mathrm{B}\mathrm{m}}\right)\frac{{N}_{\mathrm{B}\mathrm{m}}}{{K}_{\mathrm{B}\mathrm{m}}}\right){N}_{\mathrm{B}\mathrm{m}}-{\lambda}_{MBm}{S}_{\mathrm{B}\mathrm{m}}-{m}_{\mathrm{B}\mathrm{m}}{S}_{\mathrm{B}\mathrm{m}}\\ \frac{\mathrm{d}{E}_{\mathrm{B}\mathrm{m}}}{\mathrm{d}t}={\lambda}_{MBm}{S}_{\mathrm{B}\mathrm{m}}-{\gamma}_{\mathrm{B}\mathrm{m}}{E}_{\mathrm{B}\mathrm{m}}-{m}_{\mathrm{B}\mathrm{m}}{E}_{\mathrm{B}\mathrm{m}}\\ \frac{\mathrm{d}{I}_{\mathrm{B}\mathrm{m}}}{\mathrm{d}t}={\gamma}_{\mathrm{B}\mathrm{m}}{E}_{\mathrm{B}\mathrm{m}}-{\alpha}_{\mathrm{B}\mathrm{m}}{I}_{\mathrm{B}\mathrm{m}}-{m}_{\mathrm{B}\mathrm{m}}{I}_{\mathrm{B}\mathrm{m}}\\ \frac{\mathrm{d}{R}_{\mathrm{B}\mathrm{m}}}{\mathrm{d}t}=\left(1-{v}_{\mathrm{B}\mathrm{m}}\right){\alpha}_{\mathrm{B}\mathrm{m}}{I}_{\mathrm{B}\mathrm{m}}-{m}_{\mathrm{B}\mathrm{m}}{R}_{\mathrm{B}\mathrm{m}}\\ \frac{\mathrm{d}{D}_{\mathrm{B}\mathrm{m}}}{\mathrm{d}t}={v}_{\mathrm{B}\mathrm{m}}{\alpha}_{\mathrm{B}\mathrm{m}}{I}_{\mathrm{B}\mathrm{m}}\end{array}$$

All other processes involved in the model setup were maintained as presented in the WNV epidemic model by Mbaoma et al.^27^. Infection and cross-infection between vector and host population were maintained in a natural cycle and the model was forced externally by temperature, precipitation, and humidity. The process was described in Eq. (13–17) representing force of infection for pathogen transmission from mosquitoes to residential birds (${\lambda}_{MBr}$), mosquitoes to migratory birds (${\lambda}_{MBm}$), mosquitoes to humans (${\lambda}_{MH}$), residential birds to mosquitoes (${\lambda}_{BrM}$), and migratory birds to mosquitoes(${\lambda}_{BmM}$).

13 $${\lambda}_{MBr}={\delta}_{M}{F}_{b}k{p}_{MBr}{\phi}_{Br}\frac{{I}_{M}}{{k}_{Mv}}$$

14 $${\lambda}_{MBm}={\delta}_{M}{F}_{b}k{p}_{MBm}{\phi}_{Bm}\frac{{I}_{M}}{{k}_{Mv}}$$

15 $${\lambda}_{MH}={\delta}_{M}{F}_{h}k{p}_{MH}{\phi}_{Bm}\frac{{I}_{M}}{{k}_{Mv}}$$

16 $$\begin{array}{c}{\lambda}_{BrM}={\delta}_{M}{F}_{b}k{p}_{BrM}\frac{{I}_{Br}}{{K}_{Br}}\\ \end{array}$$

17 $${\lambda}_{BmM}={\delta}_{M}{F}_{b}k{p}_{BmM}\frac{{I}_{Bm}}{{K}_{Bm}}$$

### Model parameters

State variables used for model initialization were presented in (Table 1). Parameters explaining transition rates of susceptible juvenile (${S}_{\mathrm{J}}$), infected juvenile (${I}_{\mathrm{J}}$) and VT rates (${J}_{\mathrm{v}}$) were added to the parameters earlier implemented by Mbaoma et al.^27^. Additionally, all other parameters and functions used in the model are contained in (Tables 2, Table 3, Table 4, Table 5).

**Table 3:** Climate dependent mosquito population parameters defined by functions. Per unit capita rates are in unit days.

Variable	Description	Function
${f}_{E}$	Transition rate from egg to larvae	$0.16\left({e}^{0.105\left(\mathrm{T}-10\right)}\right)-{e}^{0.105\left(35-10\right)-(35-T)/5.007}$*(*Rhnorm*)
${f}_{L}$	Transition rate from larvae to pupae	${f}_{p}$/4
${f}_{P}$	Transition rate from pupae to emerging adults	$0.021\left({e}^{0.162\left(\mathrm{T}-10\right)}\right)-{e}^{0.162\left(35-10\right)-(35-T)/5.007}$*1 + *Pnorm*
${f}_{Ag}$	Transition from engorged to ovipositing adults	$\left(T-{T}_{Ag}\right)/TD{D}_{Ag} if T>{T}_{Ag}$
${f}_{Ao}$	Transition rate from ovipositing to host-seeking adults	${\gamma}_{Ao}$*(1 + *Pnorm*)
${m}_{L}$	Larvae mortality rate	${\mu}_{L}+{e}^{-T/2}$
${m}_{P}$	Pupae mortality rate	${\mu}_{p}+{e}^{-T/2}$
${m}_{A}$	Adult mortality rate	-0.05941 + 0.002965 T
${m}_{DR}$	Juvenile mosquito development rate	3.76*10^–5^ $T\left(t\right)$($T\left(t\right)$ – 7.8) (38.5- $T\left(t\right)$)
${P}_{LA}$	Juvenile mosquito survival rate	3.60*10^–3^ ($T\left(t\right)$ – 0.1) ( $T\left(t\right)$ –38.4)
${k}_{Lv}$	Modulated carrying capacity larvae	A*${k}_{Lf}*Rh$
${k}_{Pv}$	Modulated carrying capacity pupae	A*${k}_{Pf}*Rh$
${k}_{Mv}$	Modulated carrying capacity mosquitoes	A*${k}_{Mf}*Rh$

*A* is the area of study location, *Pnorm* is normalized daily precipitation with ranges between 0 and 1, *Rhnorm* is normalized humidity with ranges between 0 and 1.

**Table 4:** Transmission determinants defined by functions. Per unit capita rates are in unit days.

Parameter	Function
$\kappa$	$\frac{0.344}{1+1.231\mathrm{e}(-0.184(T-20))}$
${\gamma}_{M}$	$0.0093T-0.1352$
${\phi}_{Br}$	$\frac{{S}_{\mathrm{B}\mathrm{r}}}{{k}_{Mv}}$
${\phi}_{Bm}$	$\frac{{S}_{\mathrm{B}\mathrm{m}}}{{k}_{Mv}}$
${\phi}_{H}$	$\frac{{S}_{H}}{{k}_{Mv}}$

**Table 5:** Periodic functions with exponential decay describing the behavior of chemical and larvicides used in chemical and biological control respectively estimated from Carvalho et al.^30^.

Variable	Description	Function
${\mu}_{A,}^{^{\prime}0}{ e}^{-kt}$	Decay function for insecticide	$0.958{e}^{-0.5t}$
${\mu}_{L,}^{^{\prime}0}{ e}^{-kt}$	Decay function for larvicide	$0.825{e}^{-0.5t}$

The number of active mosquitoes was modulated by diapause which is a proxy for proportion of active mosquitoes defined by daytime length. The equation for diapause was defined in Eq. (18–20).

18 $${\delta}_{M}=1-\frac{1}{1+1775.7\mathrm{e}\mathrm{x}\mathrm{p}\left[1.559\left(D-18.177\right)\right]}$$

where *D* in Eq. 9 is defined as:

19 $$D=7.639arcsin\left[tan\left(\epsilon \right)tan\left(\varphi \right)+\frac{0.0146}{cos\left(\epsilon \right)cos\left(\varphi \right)}\right]+12$$

And $\epsilon$ in Eq. 10 defined as:

20 $$\epsilon =0.409sin\left(\frac{2\pi \left(d-80\right)}{365}\right)$$

${\delta}_{M}$ represents the length of daylight time in hours normalized between 0 and 1.

Basic reproductive number (R_0_) which represents the number of secondary infections that will occur from the introduction of a single infectious mosquito to a susceptible population where baseline conditions are met was computed with a similar ODE applied in the WNV epidemic model and shown in Eq. (21). ^27^

21 $$R_{0} = \sqrt {\left[ {\frac{{\gamma_{M} \beta_{Mbr} + \beta_{Mbm} }}{{\left( {\gamma_{M} + m_{M} } \right)m_{M} }} \frac{{S_{B} }}{{K_{Br} }} + \frac{{S_{B} }}{{K_{Bm} }}} \right]\left[ {\left[ {\frac{{\gamma_{Br} \beta_{Br} }}{{\left( {\gamma_{Br} + m_{Br} } \right)\left( {\alpha_{Br} + m_{Br} } \right)}}\frac{{S_{M} }}{{K_{Br} }}} \right] + \left[ {\frac{{\gamma_{Bm} \beta_{Bm} }}{{\left( {\gamma_{Bm} + m_{Bm} } \right)\left( {\alpha_{Bm} + m_{Bm} } \right)}}\frac{{S_{M} }}{{K_{Bm} }}} \right]} \right]}$$

### Vertical transmission impact rate

The impact of VT was calculated using a simple normalized difference approach. This was done using the min–max normalization approach where the original values are normalized to range between 0 and 1^66^ as shown in Eq. (22).

22 $$x^{\prime}=\left(\frac{x-min(\mathrm{x})}{max\left(\mathrm{x}\right)-min(\mathrm{x})}\right)$$

First, the raw difference between simulated WNV infection risk with VT and WNV infection risk without VT ($x$) is obtained. This difference ($x$) is then normalized using the min–max normalization approach to obtain the VT impact rate ($x^{\prime})$.

### Control efficiency analysis

It is imperative to perform efficiency analysis for each control strategy applied in an epidemiological model where control strategy has been implemented. This enables us to provide information on the best control strategy^30,31^. In this study, we compared the effects of different control strategies on the number of infected host (infected birds and infected humans) through WNV infection outbreak by the initiation of an efficiency index denoted by ${\mathbb{F}}$. Generally, efficiency index was defined as shown in Eq. (23).

23 $${\mathbb{F}}=\left(1-\frac{{A}_{v}^{c}}{{A}_{v}^{0}}\right)\times 100$$

where ${A}_{v}^{c}$ represents the area under the curve for total infected host population between the time interval [0, t] when the controls are implemented. ${A}_{v}^{0}$ is the area under the curve for total infected host population during the time interval [0, t] when no control method was used.

### Model assumptions and initialization

Generally, the model assumes that a small number of infected mosquitoes (${(I}_{\mathrm{M}}$) are introduced into a disease-free population of mosquitoes. Subsequently the population of these mosquitoes respond to the conditions of their immediate environment represented by several functional traits including development rate, mortality rate, host-seeking rate, ovipositing rate and pathogen development rate. Although young and older gravid adult (${(A}_{1g},{ A}_{2\mathrm{g}}$) mosquitoes go host-seeking and feed on disease-free birds, only older gravid mosquitoes (${A}_{2\mathrm{g}}$) deposit infectious substances as they probe through their host’s skin. Older ovipositing infected mosquitoes (${A}_{2\mathrm{o}}$) lay infected eggs at a rate (${J}_{v}$) which supplements the active transmission cycle and alters the epidemiology of WNV transmission in a location. Disease-free mosquitoes which go host-seeking for blood meals might also become infected after taking up blood meal from an infected bird which are amplifying, or reservoir hosts of WNV. *Culex* mosquitoes which also feed on other mammals may take up blood meals from humans and horses, depositing infected substances as they probe the skin for blood. In contrast, mosquitoes do not get infected from feeding on humans and horses because they are considered dead-end hosts. Infection and cross-infection continue, and transmission is maintained in an enzootic cycle between *Culex* mosquitoes and competent birds. Migratory birds which become infected may introduce the virus to new locations along their migratory path.

We initialized the model using parameters and functions that describe mosquito population and pathogen transmission, with a spatial extent covering Germany and temporal extent between 2018 and 2023. All state variables were set at 0, excluding mosquito eggs (E), carrying capacity of residential birds (${K}_{Br}$), carrying capacity of migratory birds (${K}_{Bm}$), susceptible residential bird (${S}_{Br}$), and susceptible migratory birds (${S}_{Bm}$). We assumed that only 10% of each bird category were susceptible and exposed to WNV infection at a given time due to the nature of bird movement. Carrying capacity of migratory and residential birds were obtained from bird observation for Germany as seen on E-bird website^64^. Bayesian estimation method was used to for inverse calibration after sensitivity analysis was performed to identify the effect parameters with the most effect on WNV infection risk. The calibration process has been documented in the precursor model^27^. All analyses and visualizations were performed in R (version 4.3.0)^67^.

## Supplementary Information

Supplementary Information.

## Data Availability

All data used for this research is publicly available and relevant references have been provided to all data sources in-text.
