# Supplementary material for: Incorporating vertical transmission into mechanistic modeling of West Nile virus for optimized control in Germany
Source: Sci Rep. 2026 Jul 18;16:22547. doi: 10.1038/s41598-026-58371-8 (PMC13380618; doi:10.1038/s41598-026-58371-8)
Supplement: Supplementary file 1 — Supplementary Information. [file 41598_2026_58371_MOESM1_ESM.docx]

**Supplementary Information 1 Results**

Simulated West Nile virus infection risk maps for model with vertical transmission across Germany between 2018 and 2023

**With mechanical control**

**Without control**

**With biological control**

**With chemical control**

**
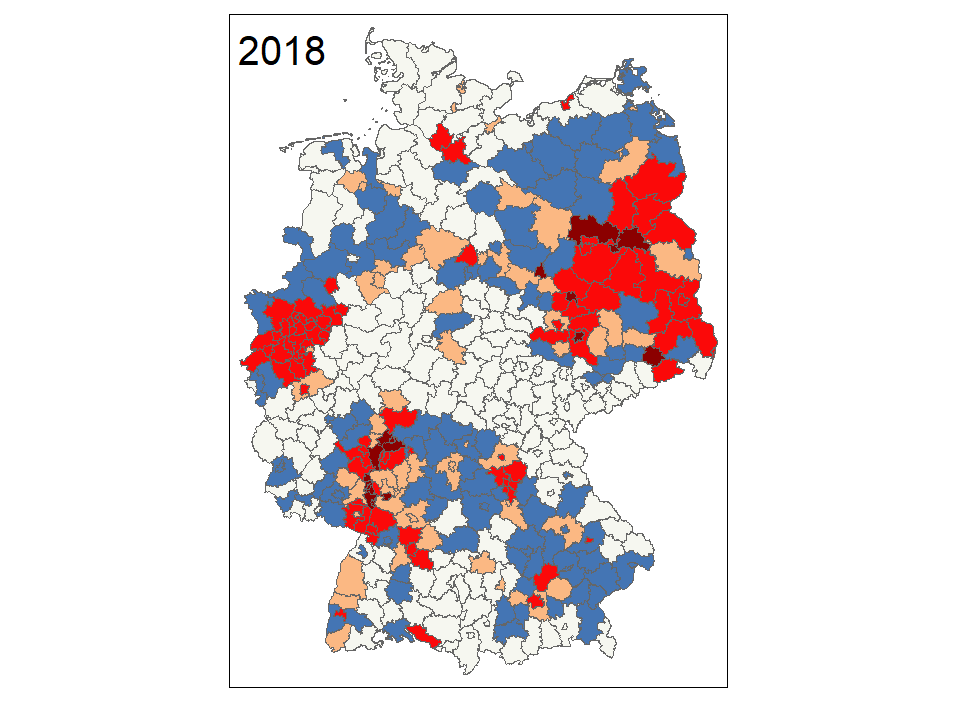
**

**
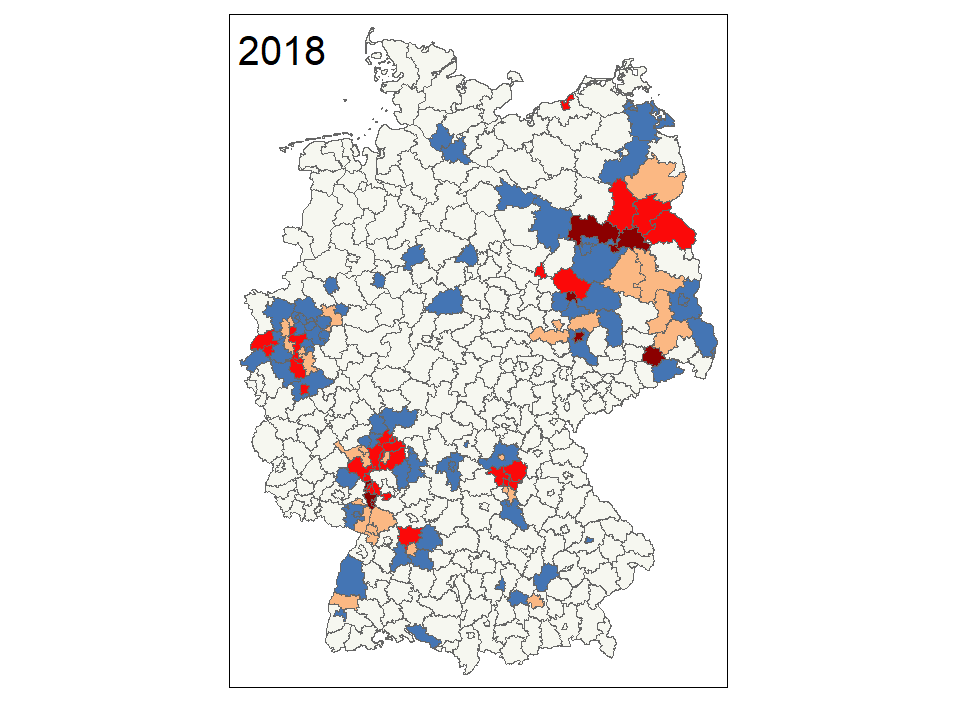

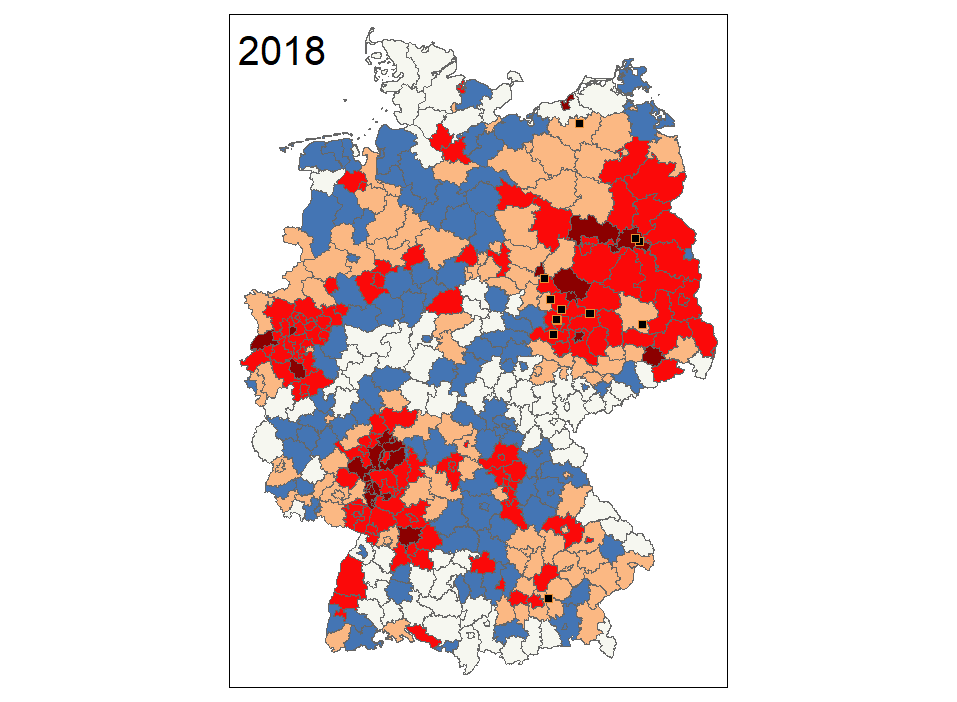

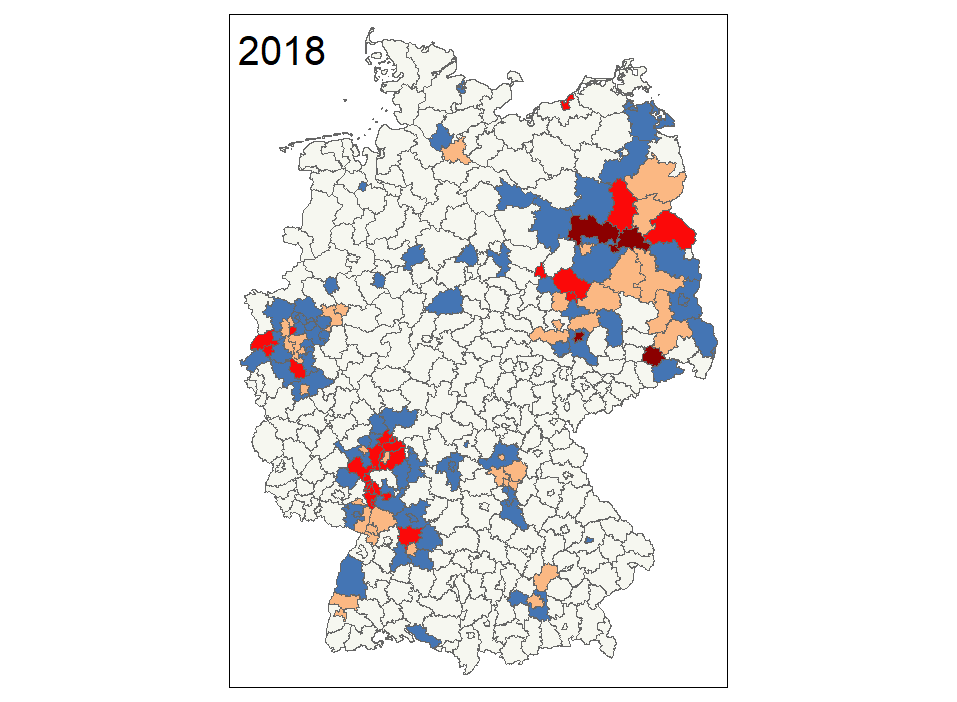

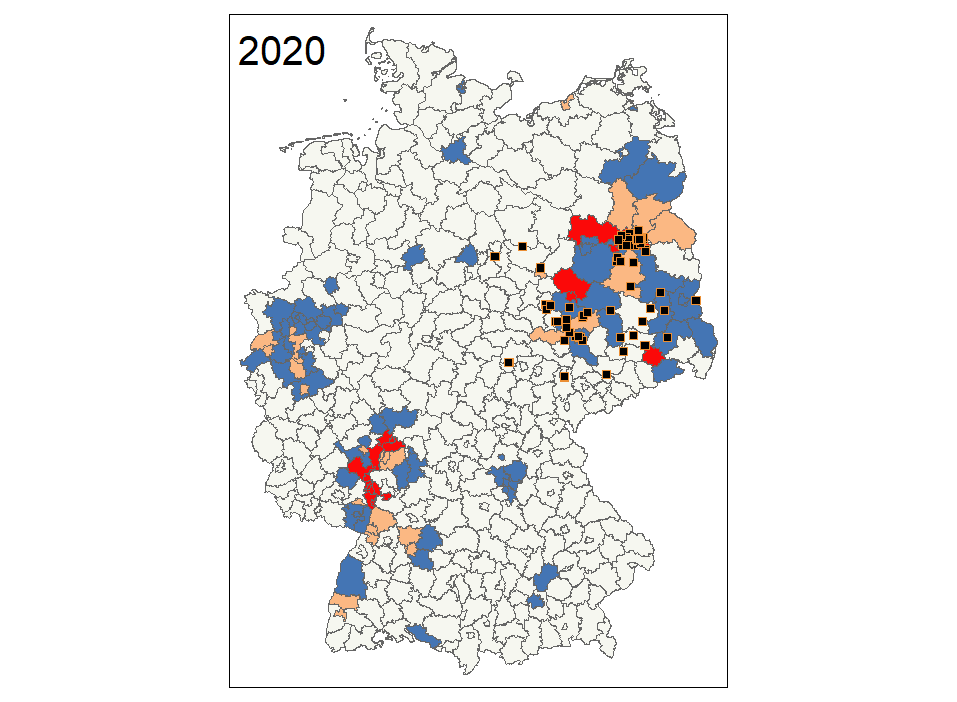

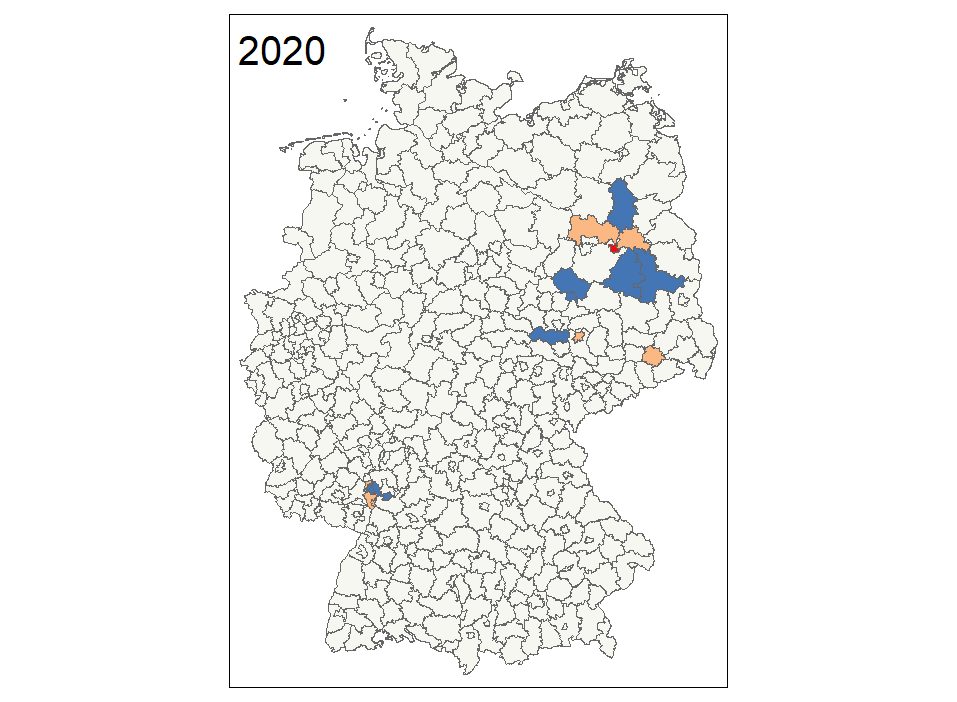
**

**2018**

**
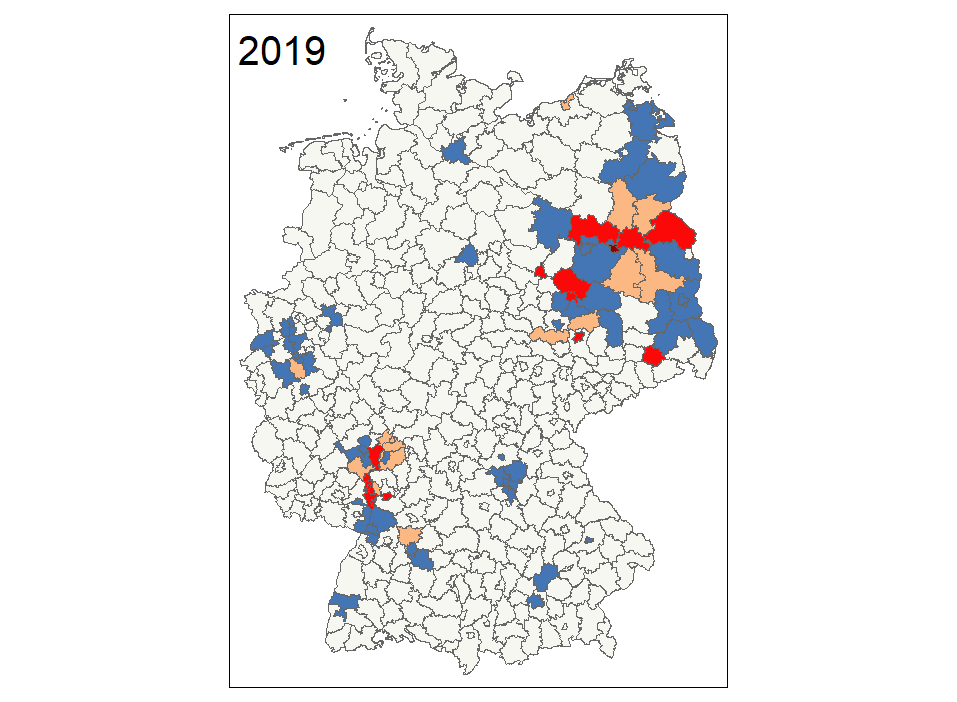

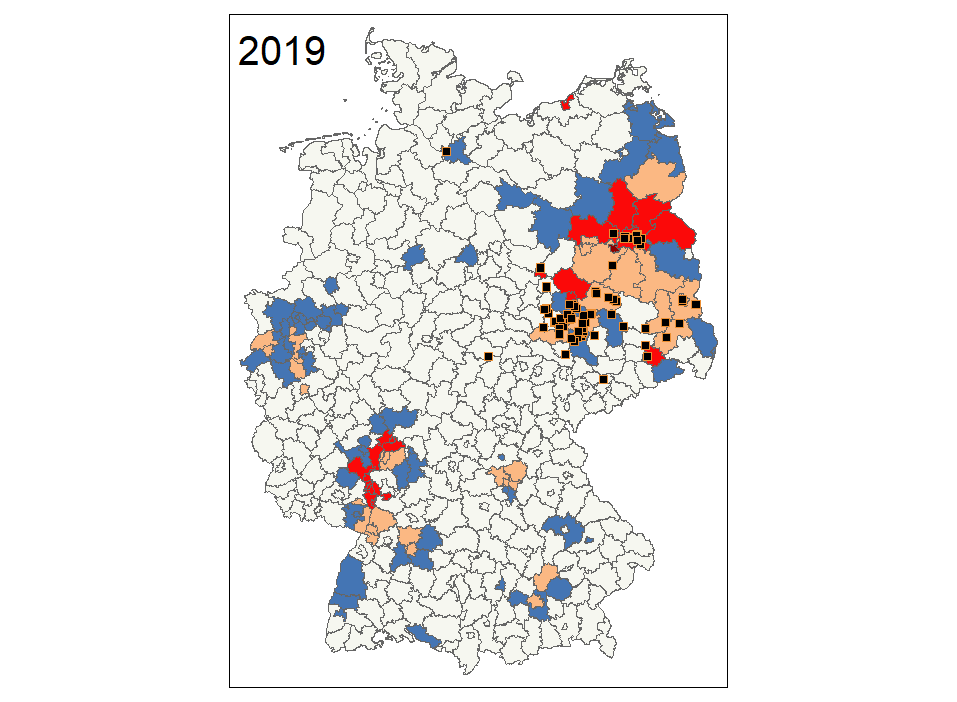

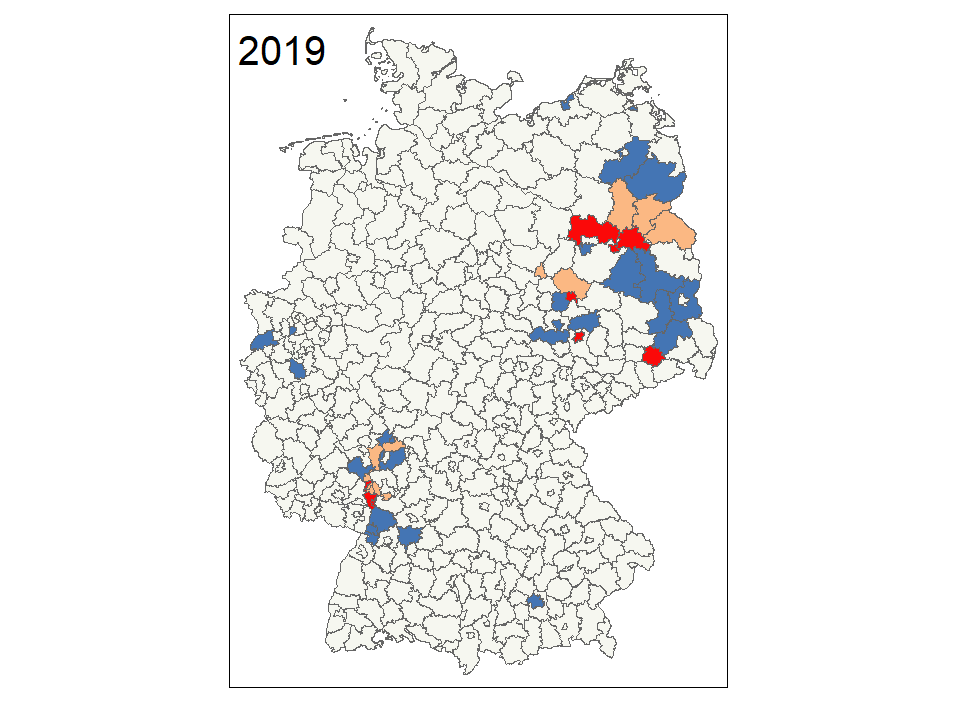

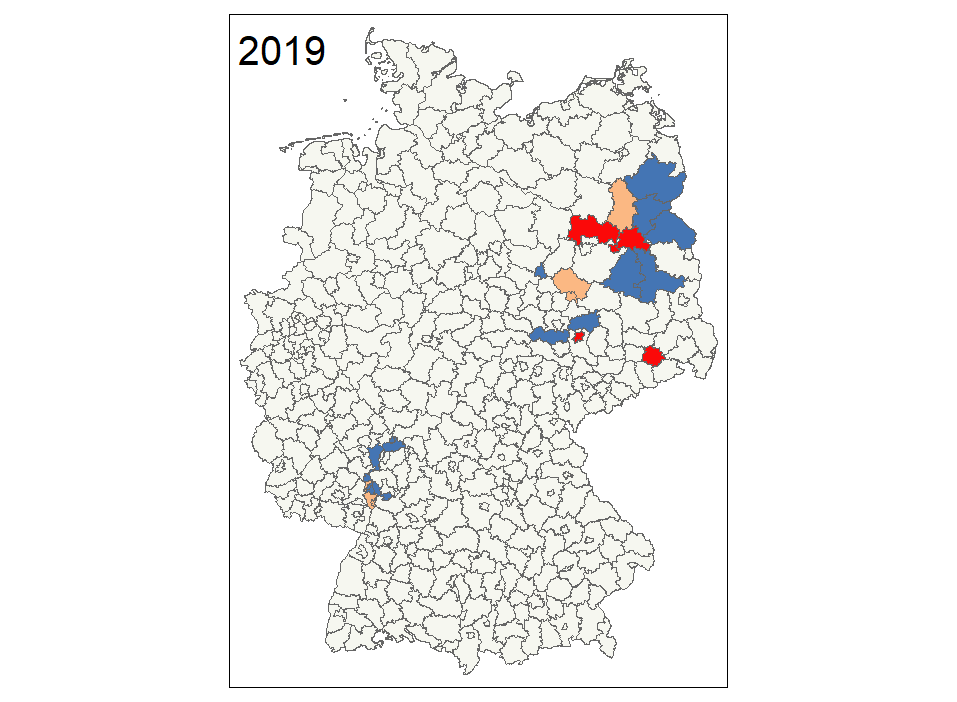
**

**2019**

**
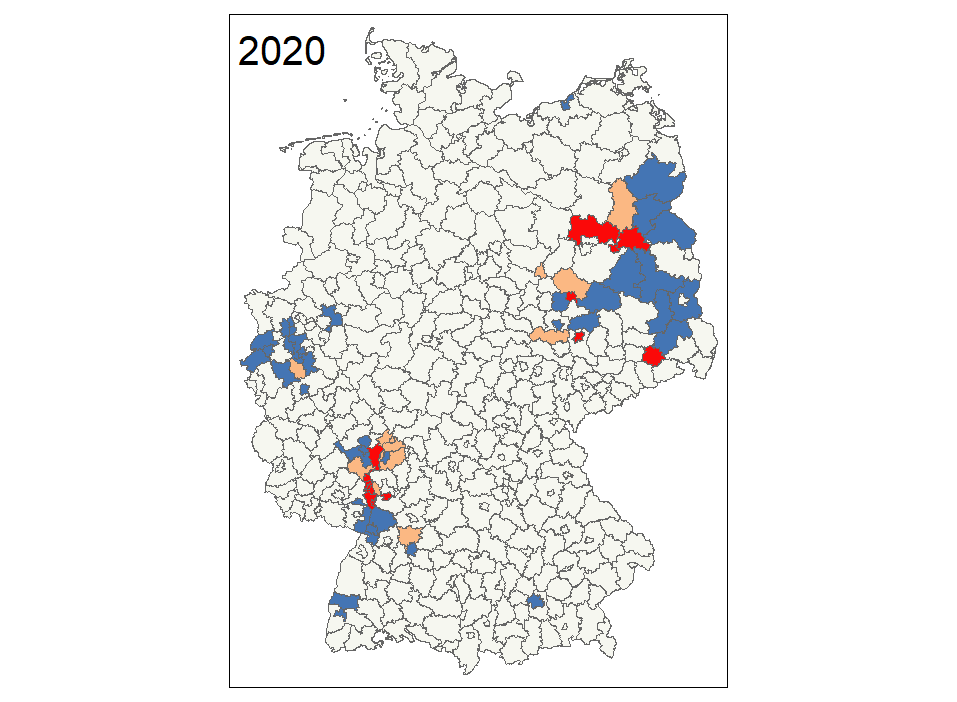

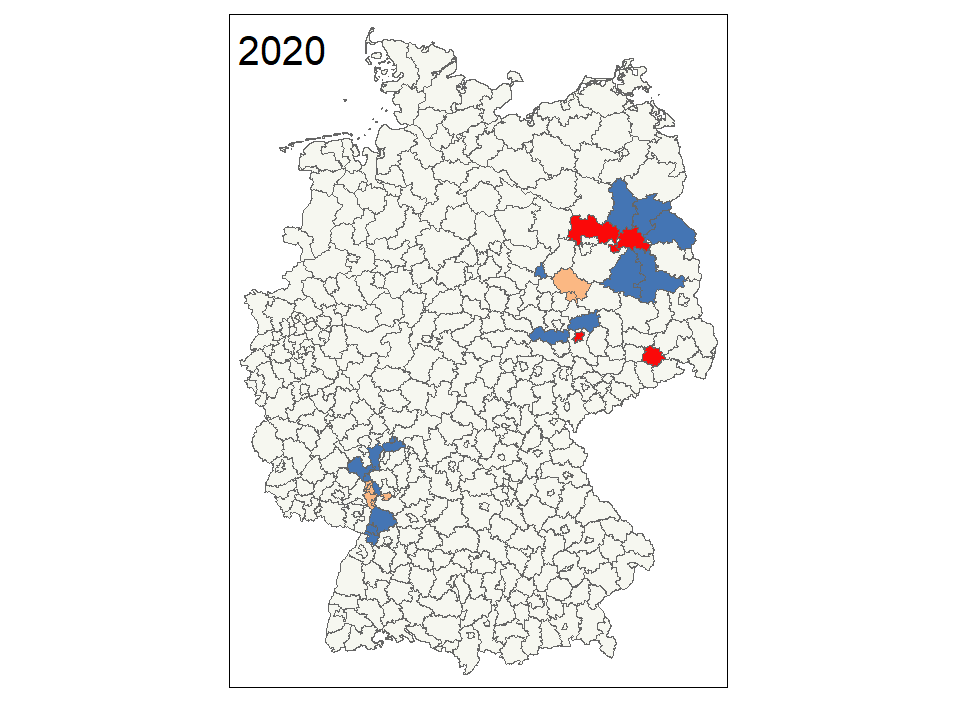
**

**2020**

**
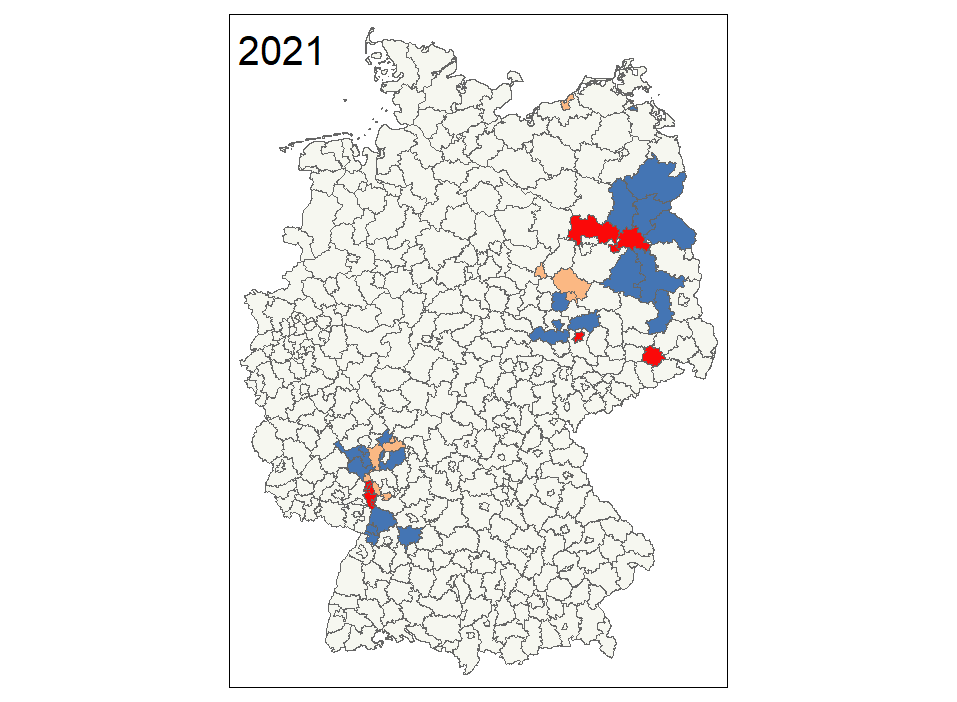

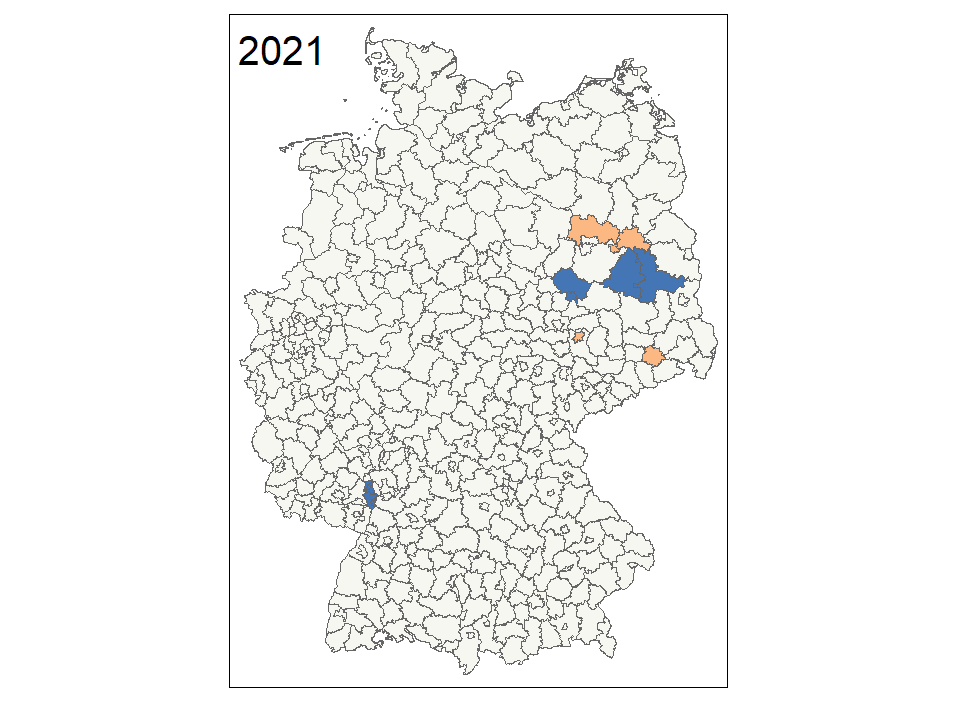

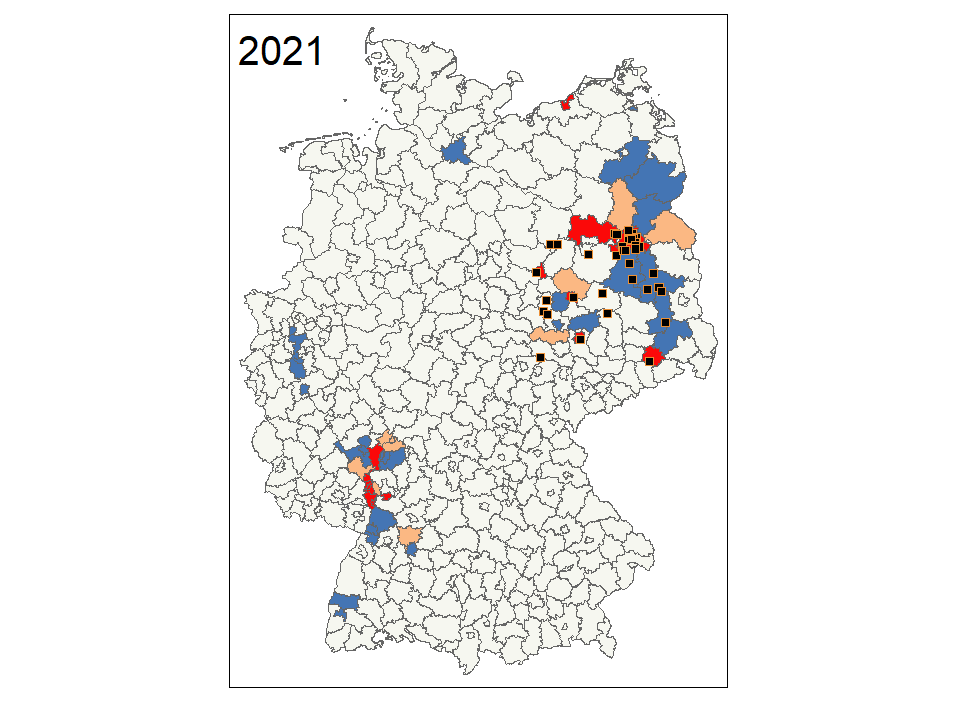

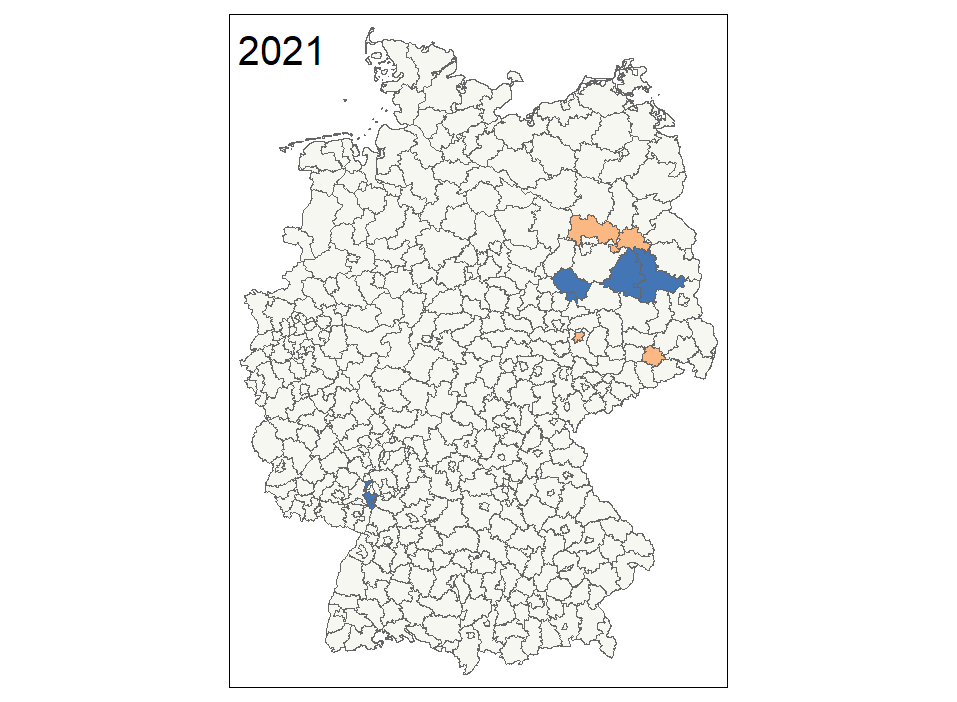
**

**2021**

**
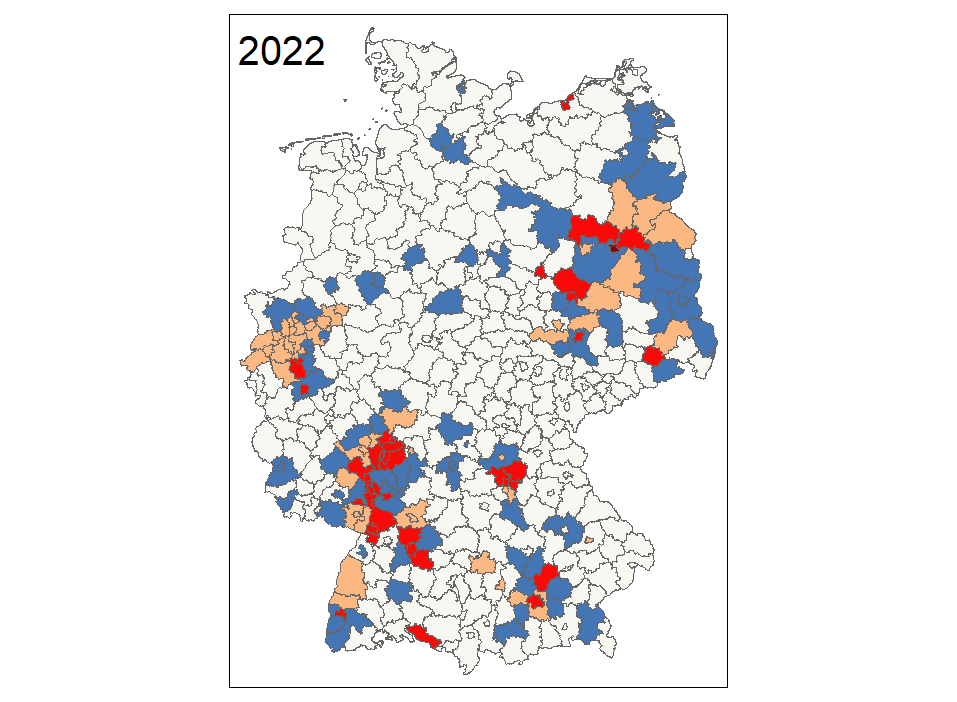

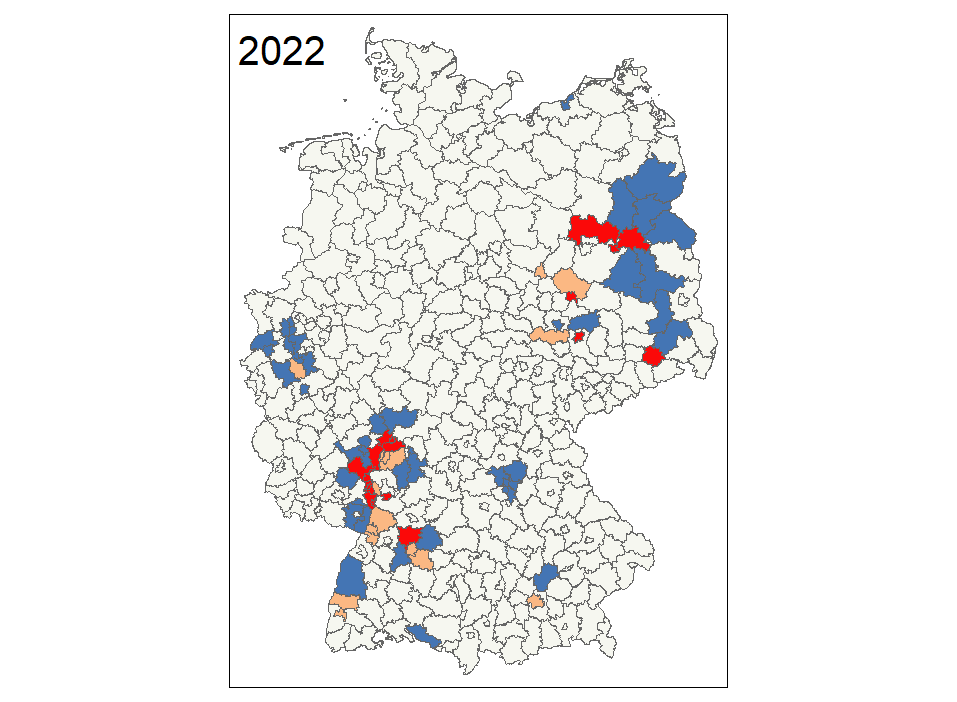

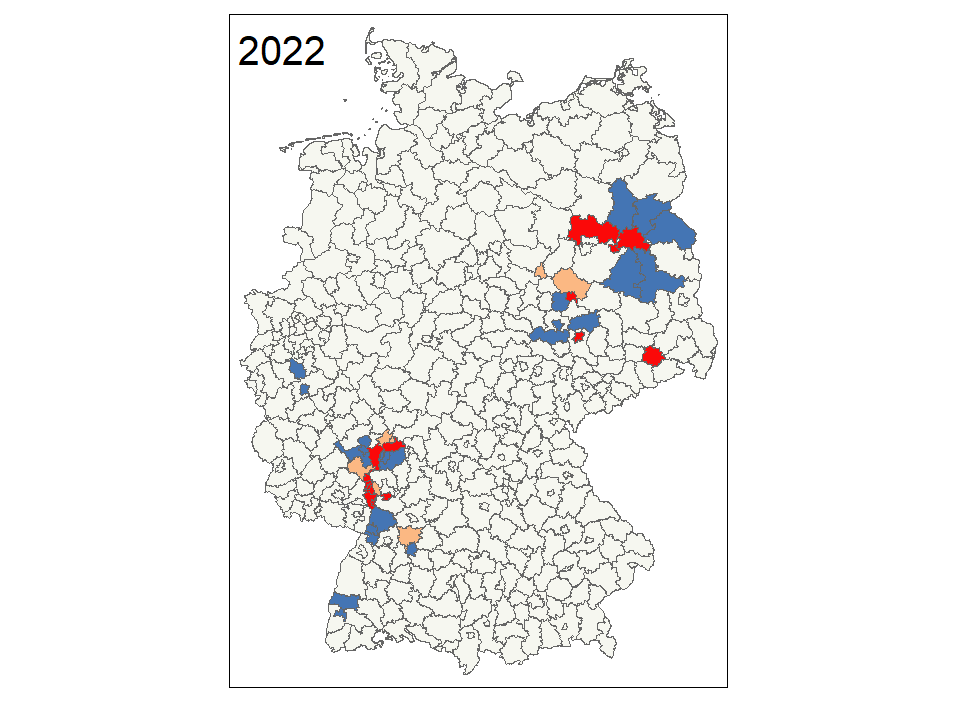

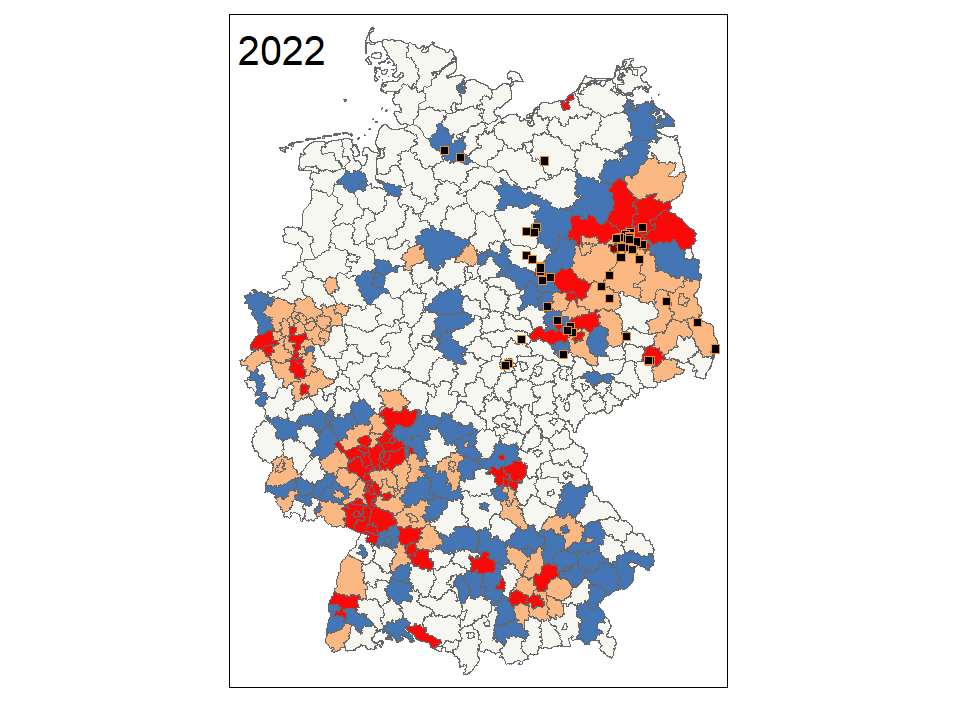
**

**2022**

**
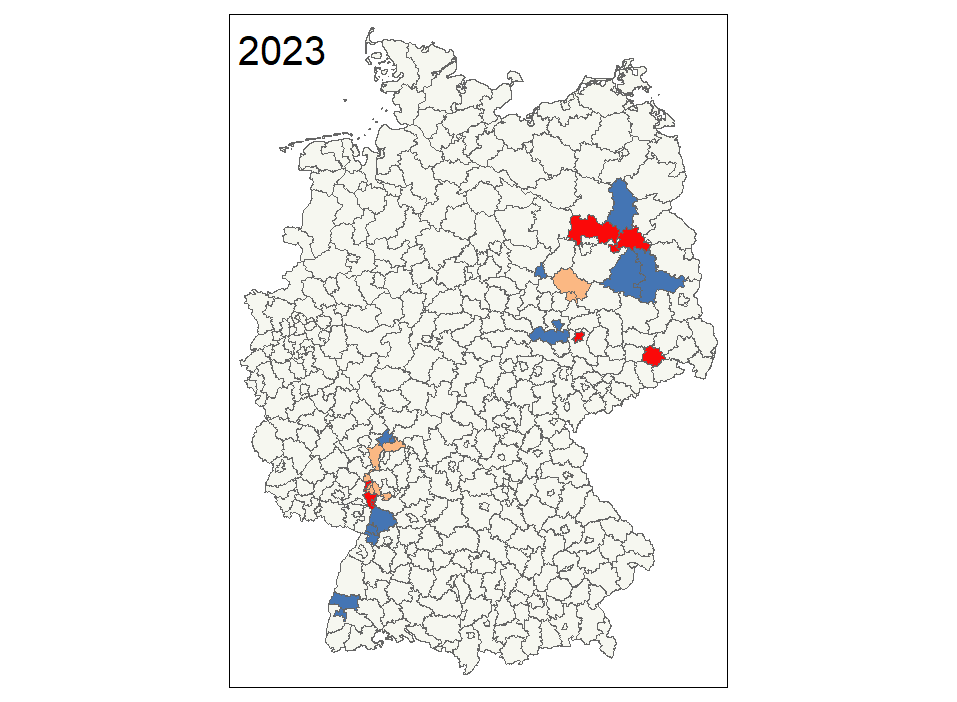

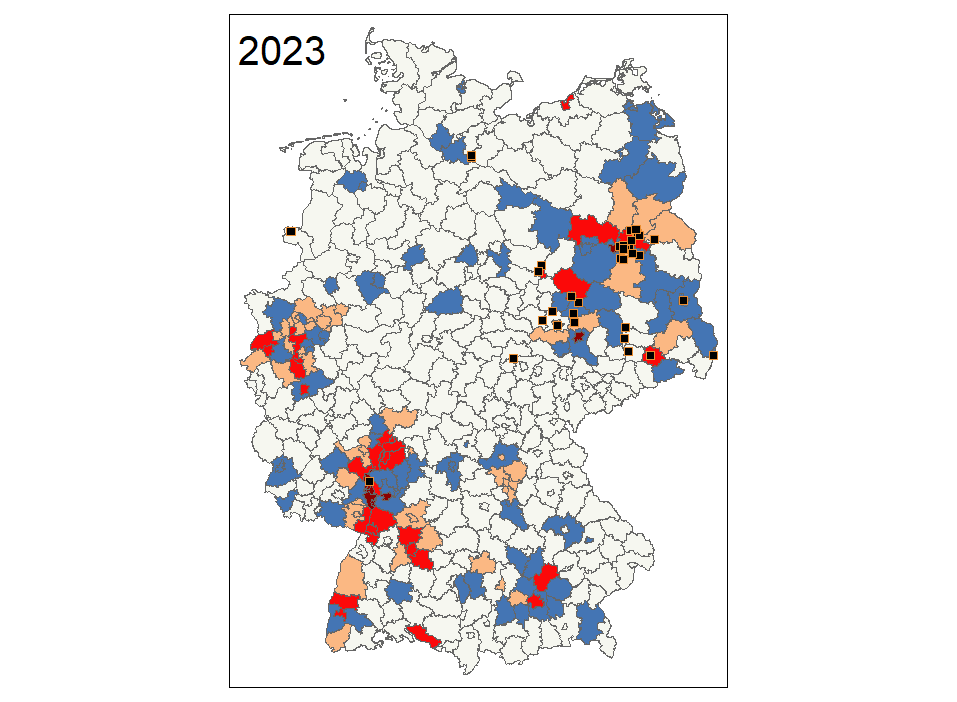

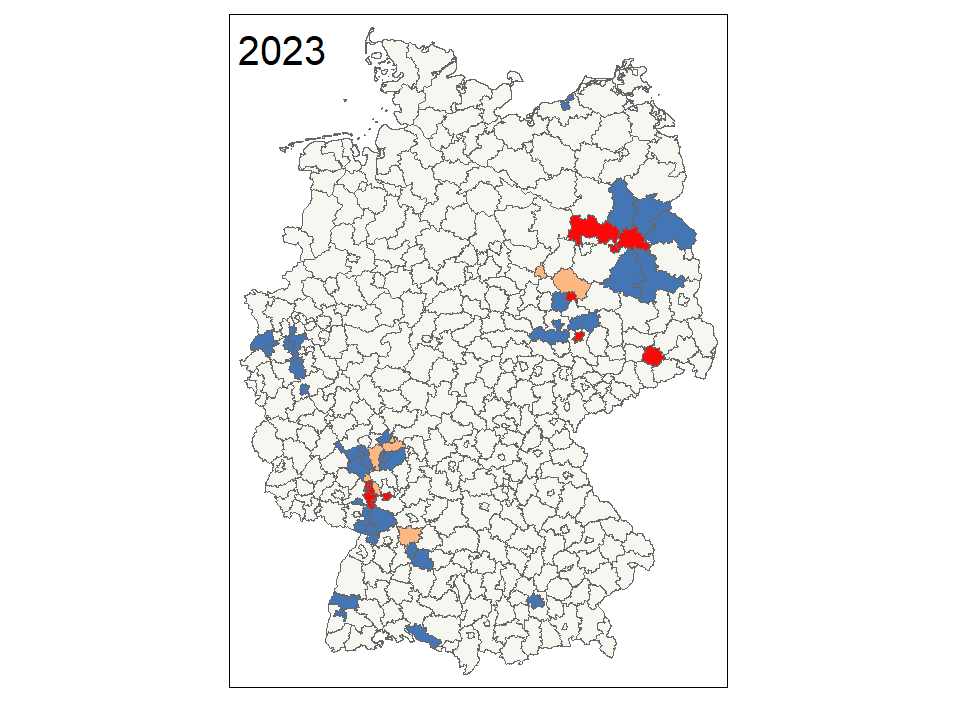

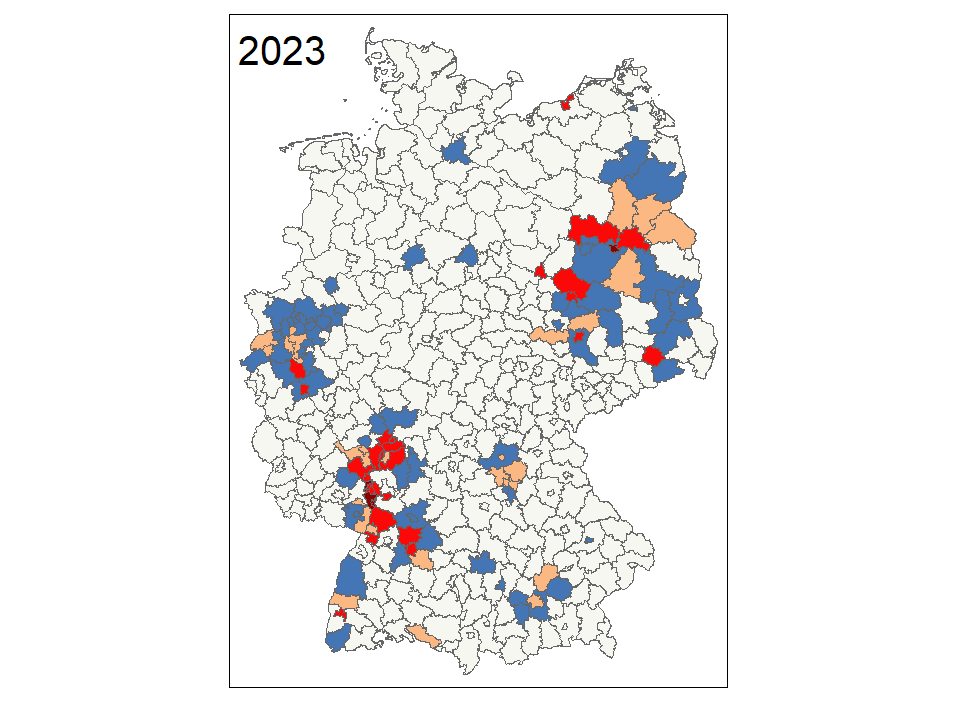
**

**2023**


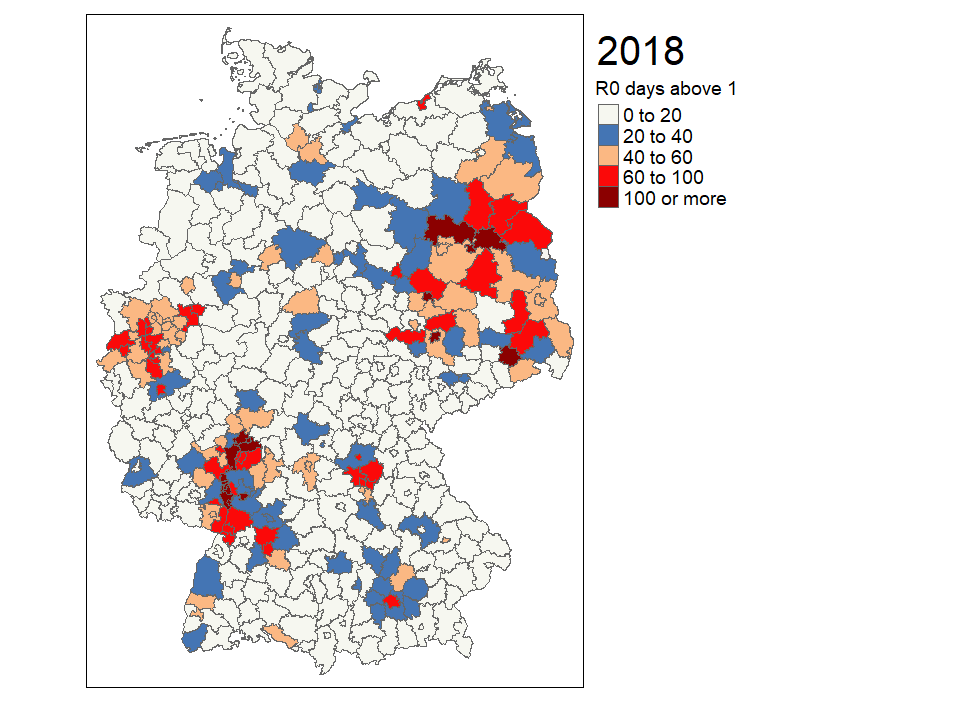


**Figure** S1 Simulated West Nile virus infection risk maps for model with vertical transmission across Germany between 2018 and 2023.
